# Cerebrospinal Fluid-Derived Small Extracellular Vesicles May Better Reflect Medulloblastoma Proteomes than Those from Blood Plasma

**DOI:** 10.3390/ijms26199279

**Published:** 2025-09-23

**Authors:** Laura Reetz, Jamal Ghanam, Venkatesh K. Chetty, Lennart Barthel, Stephan Tippelt, Gudrun Fleischhack, Marie Böckmann, Katarina Reinhardt, Basant K. Thakur

**Affiliations:** 1 Department of Pediatrics III, University Hospital Essen, Hufelandstrasse 55, 45147 Essen, Germany; laura.reetz@uk-essen.de (L.R.);; 2 Department of Gastroenterology, Hepatology and Transplant Medicine, University Hospital Essen, Hufelandstrasse 55, 45147 Essen, Germany; 3 Department of General, Visceral, Vascular and Transplant Surgery, Medical Faculty, University Hospital Essen, Hufelandstrasse 55, 45147 Essen, Germany; 4 Department of Neurosurgery and Spine Surgery, Center for Translational Neuro- and Behavioral Sciences, University Hospital Essen, Hufelandstraße 55, 45147 Essen, Germany; 5 Pediatric Oncology and Hematology, Department of Pediatrics III, University Hospital Essen, 45147 Essen, Germany

**Keywords:** small extracellular vesicles, liquid biopsy, cerebrospinal fluid, blood plasma, pediatric central nervous system tumors, medulloblastoma, EV proteomics

## Abstract

The understanding of medulloblastoma (MB) progression is limited by the lack of minimally invasive monitoring methods. Extracellular vesicles (EVs) carrying disease-specific signatures are promising for liquid biopsies, but clinical translation is hindered by inconsistent isolation techniques. This study compares small EVs (sEVs) and their proteomes from blood plasma (BP) and cerebrospinal fluid (CSF) in MB. Using ultrafiltration and size exclusion chromatography (UF-SEC), we isolated sEVs from pediatric patient samples. sEV proteins from matched CSF-BP samples from MB patients (MBCSF/MBBP), healthy BP controls (HCBP), and MB cell lines (MBCL) were analyzed by liquid chromatography-tandem mass spectrometry, subjected to Gene Ontology and Cytoscape analyses, and compared to published MB, CSF, and EV datasets. By optimizing UF-SEC for small volumes, we found that CSF-sEVs are smaller and elute in later SEC fractions. Proteins linked to the extracellular matrix (ECM) were enriched in MBCSF and MBCL, while integrin binding showed inconsistent patterns between MBCSF and MBBP. MBBP and HCBP showed no significant differences. Fourteen proteins from MB datasets were identified in our analysis and primarily enriched in CSF. These findings support CSF-sEVs as more informative than BP-sEVs for MB diagnosis and monitoring, emphasize the need for fluid-specific sEV isolation, and suggest that ECM components and integrins may mediate MB progression.

## 1. Introduction

Medulloblastoma (MB) is the most common malignant brain tumor in pediatric patients and the leading cause of cancer-related mortality in children in the Western Hemisphere [1,2]. MB is currently classified into four subgroups based on molecular profiles (Wingless (WNT), Sonic Hedgehog (SHH), Group 3, and Group 4), which have clinical implications for prognosis and risk stratification [1,2]. However, this classification requires the acquisition of tumor tissue (tissue biopsy), which entails invasive procedures that may be complicated by the involvement of eloquent areas [3]. Therefore, minimally invasive approaches such as liquid biopsy are increasingly recognized as valuable tools for monitoring central nervous system (CNS) tumors, including MB.

Integrating tumor-derived information from body fluids could enable accurate molecular characterization for diagnosis and disease monitoring. However, the application of liquid biopsy in clinical practice has been limited by factors such as the low abundance of tumor-derived biomarkers and the short half-life and fragmentation of cell-free DNA due to degradation [4].

Small extracellular vesicles (sEVs) have emerged as promising tools for minimally invasive, dynamic disease monitoring. They are secreted by various cell types under physiological and pathological conditions and enter a variety of body fluids, including blood and cerebrospinal fluid (CSF) [4]. Their cargo, comprising cell-specific nucleic acids, proteins, and lipids, is protected from degradation by a lipid bilayer, enabling clinical application and the generation of multidimensional information in a single approach [5]. sEVs have been shown to play key roles in cell communication, tumor progression, immunomodulation, and angiogenesis [5,6,7].

EVs can cross the blood–brain barrier (BBB) via transcytosis, enabling communication between the CNS and peripheral compartments [8]. In a physiological state, the BBB is an extremely selective and semipermeable barrier of endothelial cells, regulating the transfer of chemicals and solutes between the circulatory system and the CNS [9]. However, in a pathophysiological state, the BBB is altered, and its integrity is partially compromised [9]. It has been shown that in the presence of brain tumors, the BBB is modified to form a brain tumor barrier (BTB) [10,11]. Additionally, EVs from high-grade brain gliomas have been detected in peripheral blood, underscoring their potential as liquid biopsy analytes [12,13].

While blood plasma is widely used for sEV analysis due to its ease of collection, abundant volume, and systemic distribution, specimen choice may vary depending on tumor type and disease stage [14,15]. Research on CNS tumors has centered on the use of CSF due to its close proximity to the tumors [16,17].

Currently, the diagnostic and functional potential of sEVs is limited due to the lack of a standardized sEV isolation protocol [18,19]. In addition, the available volumes vary depending on the source material. While large volumes of blood, urine, or cell culture supernatant can typically be obtained, the amount of CSF is generally limited and inconsistent due to physiological and pathological variability [20].

Moreover, individual properties of body fluids could affect sEV isolation, comprehensive studies on disease-related information and their sensitivity are limited. These factors must be considered for sEV preparation. Furthermore, studies on sEV proteins are challenging, as soluble proteins are a major contaminant in most sEV isolation methods, and their association with sEVs should be carefully evaluated [6].

This study compares the isolation of sEVs from blood plasma (BP) and CSF obtained from pediatric patients using a combination of ultrafiltration and size exclusion chromatography (UF-SEC). Furthermore, the study evaluates the eligibility and clinical utility of the respective sEV proteomes from BP and CSF, compared to healthy BP and MB cell lines, in the context of MB. As sEVs from BP and CSF appeared to differ in size, they enriched in different SEC fractions. Furthermore, our data suggested that CSF was enriched in MB-associated proteins, compared to BP. Gene Ontology (GO) and Cytoscape analyses revealed that extracellular matrix (ECM) components and integrins may play a role in MB progression.

## 2. Results

### 2.1. sEVs Elute in Different Fractions Depending on the Body Fluid

To overcome the challenge of variable CSF volumes, we used ultrafiltration followed by size exclusion chromatography (UF-SEC) to isolate sEVs from low volumes of BP and CSF samples. UF enables the concentration of varying input volumes to a standardized volume for SEC. UF-SEC has been described as an effective method for sEV isolation with high recovery rates [21,22], making it suitable for pediatric samples with limited volume. Therefore, we optimized UF-SEC for sEV isolation from pediatric BP and CSF samples and characterized them according to MISEV2023 guidelines [6].

To compare BP- and CSF-derived sEV distribution across the SEC fractions, we analyzed particle and free protein concentrations in the collected fractions F1–F10. Free proteins are one of the main contaminants in any EV preparation and are known to be abundant in later SEC fractions, while sEVs usually elute mainly in early SEC fractions. BP samples showed overall higher concentrations of both particles and free proteins than CSF did (Supplementary Figure S1A,B). However, their range across the fractions differed. Notably, CSF had peak particle concentrations in later fractions, while both fluids showed increased free protein levels in later fractions (Supplementary Figure S1A,B).

**Figure 1:**
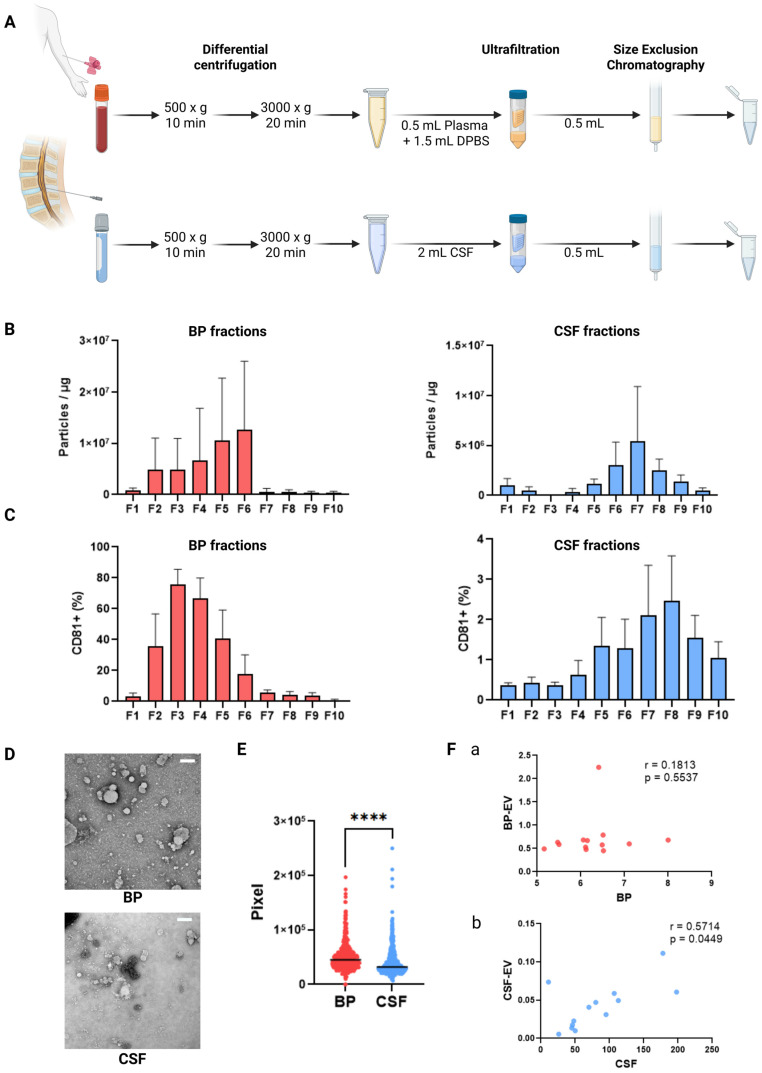
Characterization of particles and small extracellular vesicles (sEVs) from blood plasma (BP) and cerebrospinal fluid (CSF). (**A**) Schematic workflow of sEV isolation using ultrafiltration–size exclusion chromatography (UF-SEC). (**B**) Assessment of sEV purity from the ratio of particles per µg protein per fraction of BP and CSF. (**C**) Mean fluorescence intensity (MFI) of CD81+ objects per fraction from BP and CSF using semi-quantitative bead-based flow cytometry. (**D**) Representative images of negative staining transmission electron microscopy (NSTEM) of sEV-fractions from BP and CSF. (**E**) Measurement of the diameter of EV-like objects from BP and CSF (in pixel) from TEM images using the ImageJ software. (**F**) Correlation analysis between protein concentrations of total proteins and soluble, co-isolated proteins from (**a**) BP and (**b**) CSF. **** *p* < 0.0001.

The degree of purity per fraction was determined by calculating the number of particles per protein for each fraction (Figure 1B). Non-protein particle numbers from BP and CSF approximated, indicating an abundance of free proteins in BP (Figure 1B).

To identify sEV-containing fractions, all collected SEC fractions were stained for CD81. BP fractions showed considerably higher CD81 mean fluorescence intensity (MFI) folds than CSF, and CD81 signals in CSF shifted to later fractions (Figure 1C). This indicates that early CSF fractions lack sEVs, while later ones contain both sEVs and free proteins, compromising the separation typically achieved by UF-SEC. CD81-bearing sEVs were mainly found in F2–6 (BP) and F5–9 (CSF), so these fractions were pooled for further analysis based on particle concentration, particle-to-protein ratio, and CD81 MFI values. The presence of sEVs in the pooled fractions was further confirmed by TEM imaging, visualizing typical sEV shape and morphology (Figure 1D).

Since sEVs from CSF eluted in later fractions, we assessed whether they are smaller in size. Measuring the diameter of particles with sEV morphology in TEM images, we found that, on average, sEVs from CSF were indeed significantly smaller compared to BP-sEVs (Figure 1E).

We further examined whether total protein concentration measured during the diagnostic work-up correlated with free protein concentrations in the pooled sEV fractions (Figure 1F, and Supplementary Figure S1C). While no correlation was found in BP-sEVs, CSF samples demonstrated a positive correlation, suggesting the method cannot fully distinguish sEV proteins from co-isolated free proteins. However, its sensitivity to small protein variations highlights comprehensive diagnostic potential. Accordingly, we refer to all proteins in pooled sEV fractions as “sEV proteins”, regardless of their origin. Notably, no correlation was found between CSF and BP protein levels or their respective sEV fractions (Supplementary Figure S1C).

### 2.2. Analysis of BP and CSF sEV Proteome Reveals Body-Fluid-Specific Protein Ontology Enrichments

To assess the diagnostic value of sEVs from BP and CSF in MB, we compared their sEV proteomes using LC-MS/MS. We analyzed three matched BP and CSF sample pairs from MB patients alongside healthy BP controls (HCBP) and pooled sEVs from MB cell lines (DAOY, UW228, ONS76; MBCL). Protein levels in MBBP (blood plasma from MB patients), MBCSF (CSF from MB patients), and MBCL were normalized to HCBP, with MBCL serving as an MB reference.

**Figure 2:**
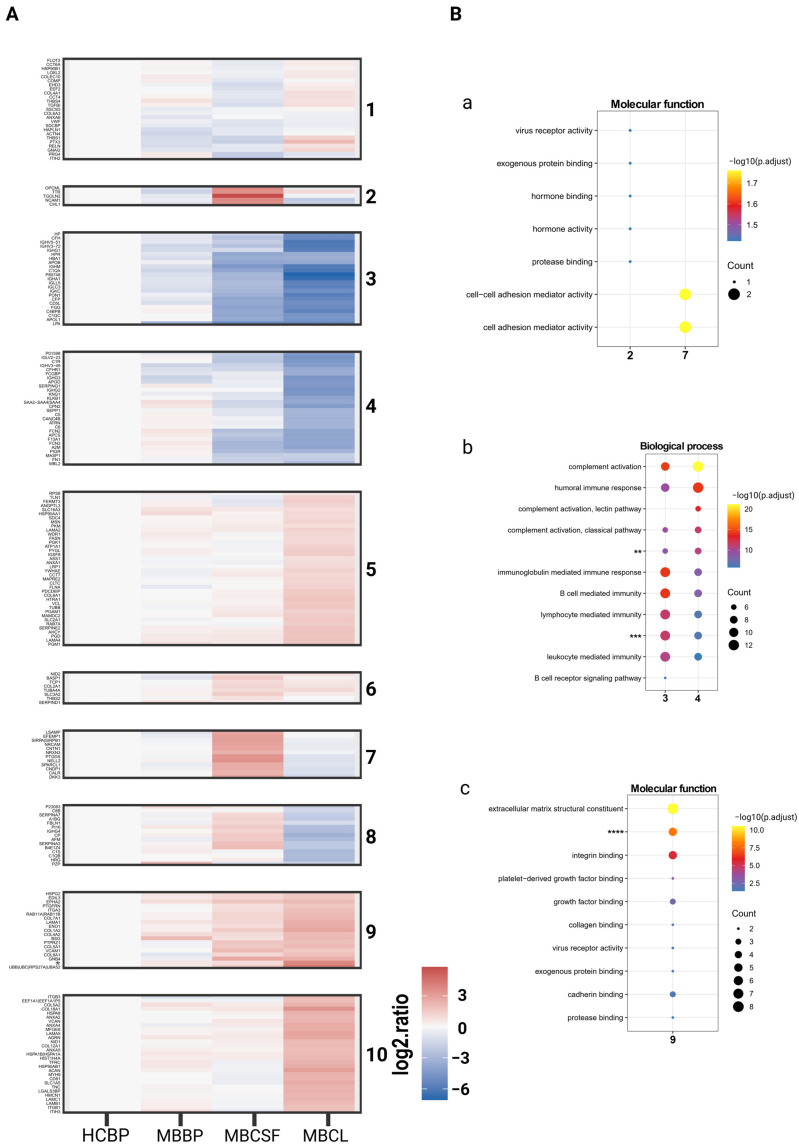
K-means clustering of 210 proteins detected by Liquid-Chromatography/Tandem Mass Spectrometry. (**A**) Heatmap presenting the proteins detected in our analysis grouped into 10 clusters. (**B**) Top 10 molecular functions (**a**,**c**) or biological processes (**b**) for proteins enriched in (**a**) cluster 2 & 7, (**b**) cluster 3 & 4, (**c**) cluster 9. * = hCG_2039566|A0A0U1RRH7|HIST1H2AB|HIST1H2AG|H2AFX|HIST1H2AD|HIST2H2AC|HIST2H2AA3|HIST3H2A|HIST1H2AC|HIST1H2AH|HIST1H2AA|HIST1H2AJ|H2AFJ, ** = humoral immune response mediated by circulating immunoglobulin. *** = adaptive immune response based on somatic recombination of immune receptors built from immunoglobulin superfamily domains, **** = extracellular matrix structural constituent conferring tensile strength.

A total of 210 unique proteins were identified and grouped into 10 clusters by K-means clustering (Figure 2A, Supplementary Figures S2–S5 and Table S2). As expected, protein abundance levels of MBBP were most similar to HCBP, while MBCSF and MBBP exhibited distinct protein patterns (Figure 2A, Supplementary Figures S2 and S6–S8). To further explore the function of the enriched proteins, each cluster was then subjected to Gene Ontology (GO) analysis to investigate associated biological processes, cellular compartments, and molecular functions. Clusters with similar patterns (2 & 7, 3 & 4, 5 & 10) were combined for the analysis.

Clusters 2 & 7, characterized by upregulation in MBCSF, were enriched for GO terms related to the nervous system, with particular enrichment for TGOLN2, a protein whose elevated expression levels have been reported in cancer and neurological disorders (Figure 2B, and Supplementary Figure S9A) [23]. Clusters 3 & 4 and 8 shared enrichment in proteins associated with the immune system and complement activation but differed in their expression patterns across the conditions, particularly in MBCSF (Figure 2B, Supplementary Figures S11B and S13). Proteins linked to the extracellular matrix (clusters 1, 5 & 10, 6, and 9) and integrin binding (clusters 1, 5 & 10, and 9) exhibited elevated levels in MBCL, and partially in MBCSF (Figure 2B, Supplementary Figures S9C and S10) had. Notably, no ontologies for molecular functions were found in cluster 6 (Supplementary Figure S11).

### 2.3. Extracellular Matrix and Integrin Binding Are Differently Expressed Between MBCL, MBCSF, and MBBP

**Figure 3:**
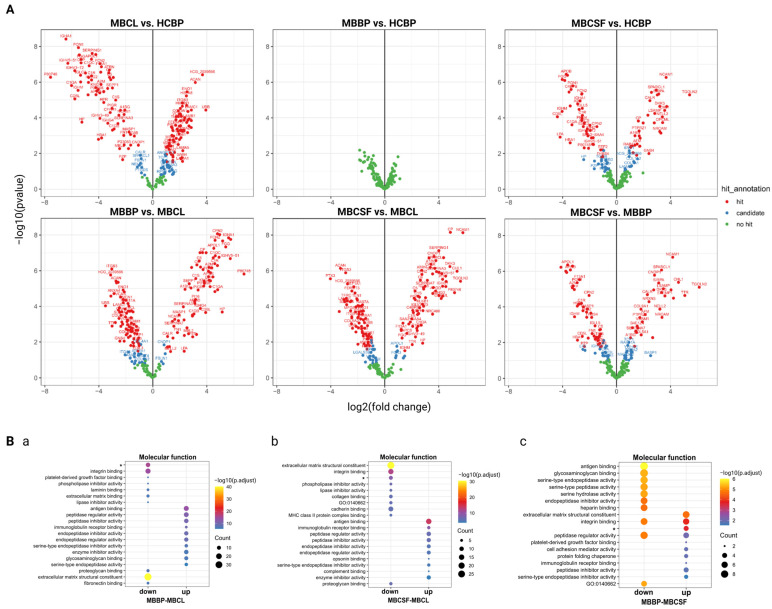
Presentation of differentially regulated proteins between MB-cell lines (MBCL), CSF from patients diagnosed with MB (MBCSF), BP from patients diagnosed with MB (MBBP) and BP from healthy individuals (HCBP). (**A**) Volcano plots illustrating differential protein level between the conditions. (**B**) Top 10 molecular functions for proteins differentially regulated between (**a**) MBCL and MBBP, (**b**) MBCL and MBCSF and (**c**) MBBP and MBCSF. * = extracellular matrix structural constituent conferring tensile strength.

Next, protein expression levels of sEVs from MBCL, MBCSF, MBBP, and HCBP were directly compared to explore similarities and differences among the conditions (Figure 3, and Supplementary Figures S15–S19). When comparing MBCL to HCBP, MBBP, and MBCSF, 178, 171, and 165 proteins were differentially regulated and classified as “hit” or “candidate”, respectively (Figure 3A). Notably, no significant differences in protein expression levels were observed between BP from MB patients and healthy controls (Figure 3A).

Comparing MBCL with the body fluids, the complement system was downregulated in MBCL, while proteins related to ECM organization and cell–matrix interactions, including integrin binding, were upregulated (Figure 3B, and Supplementary Figure S15A,B). Notably, ECM and cell–matrix organization functions were also upregulated in MBCSF compared to MBBP and HCBP (Figure 3B, Supplementary Figures S15A,B and S17). However, proteins involved in integrin binding were upregulated in both MBCSF and MBBP, suggesting a more complex or ambiguous expression pattern across the fluids (Figure 3B, and Supplementary Figure S17).

It is important to note that fewer proteins were differentially expressed, with smaller fold changes and counts in GO analyses, when comparing body fluids to each other than when comparing them to MBCL (Figure 3A,B). This observation may reflect intrinsic differences in cell line properties, such as higher metabolism and cell turnover.

### 2.4. MBBP and HCBP Differ in ECM- and Complement-Related Proteins Based on logFC Values

**Figure 4:**
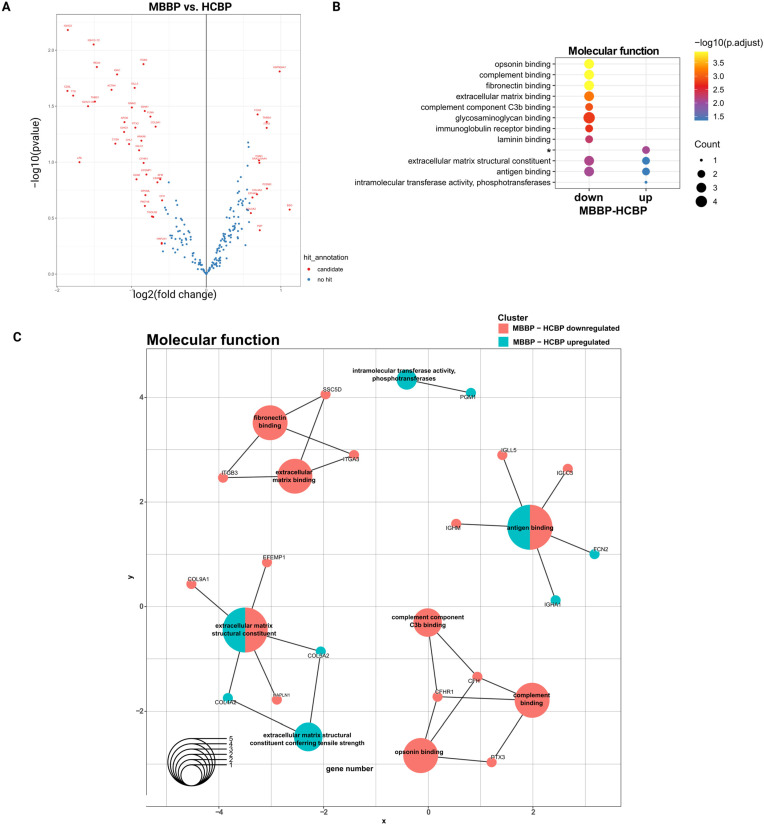
Analysis of differential regulation of proteins between MBBP and HCBP based on fold changes. (**A**) Volcano plot depicting differential protein abundance level between MBBP and HCBP. (**B**) Top 10 molecular functions for proteins differentially regulated between MBBP and HCBP. (**C**) Cnet plot of differentially regulated proteins and associated top 10 molecular functions. * = extracellular matrix conferring tensile strength.

Though no significant protein regulation differences were found between MBBP and HCBP, variations in hit annotation and GO terms were observed (Figure 3A,B, Supplementary Figures S15A,C and S16). This finding led us to hypothesize that minor changes may be masked due to the experimental setup, as BP samples from diseased and healthy individuals likely share similar proteomic profiles. While some proteins met the fold-change threshold, high FDR values excluded them as candidates. To overcome this, we reanalyzed the data with a modified hit annotation that considers only logFC values, identifying 32 new candidate proteins (Figure 4A).

Interestingly, proteins differentially expressed between MBBP and HCBP were associated with both the complement system and the extracellular matrix, in line with our previous findings (Figure 4B,C, Supplementary Figures S18 and S19). The regulation of the ECM in MBBP was complex, as ECM-related proteins were either up- or down-regulated or ambivalent to HCBP (Figure 4B,C, Supplementary Figures S18 and S19). Interestingly, proteins related to angiogenesis and integrin-mediated signaling pathways, including ITGB3, as well as those involved in complement binding were less abundant in MBBP (Figure 4B,C, Supplementary Figures S18 and S19). Moreover, some proteins abundant in MBBP were linked to the negative regulation of angiogenesis, vascular development, and blood vessel morphogenesis (Supplementary Figure S18).

### 2.5. Complement System and Integrin Cell Surface Interactions Represent Central Pathways in MB-sEVs

We further explored the enrichment of proteins for specific pathways within our dataset. Using Cytoscape (https://cytoscape.org, version 3.10.3, The Cytoscape Consortium, San Diego, CA 92107; Accessed on 8 April 2025), we analyzed all proteins in the dataset as well as those differentially regulated between two conditions. The enriched pathways were sorted based on their *p*-value.

Consistent with our findings from GO analyses, the complement system as well as beta 1 and 3 integrin cell surface interactions were among the top enriched pathways overall and across the conditions (Supplementary Table S3). Notably, integrins in angiogenesis were among the top nine pathways listed in all analyses (Supplementary Table S3).

### 2.6. CSF-sEVs Surpass BP-sEVs in Recovering MB Proteins

The large differences in protein regulation and fold changes between cell line and body fluid comparisons—likely due to cell line-specific properties—may confound true protein attributions to MB. Thus, a cell line model might not be an optimal source to identify MB-related proteins in body fluids. MBCL was therefore excluded from the analysis (Supplementary Table S4). Instead, we selected three datasets of proteins significantly expressed in MB tumor tissue (vs. healthy cerebellum) for comparison with our data.

**Figure 5:**
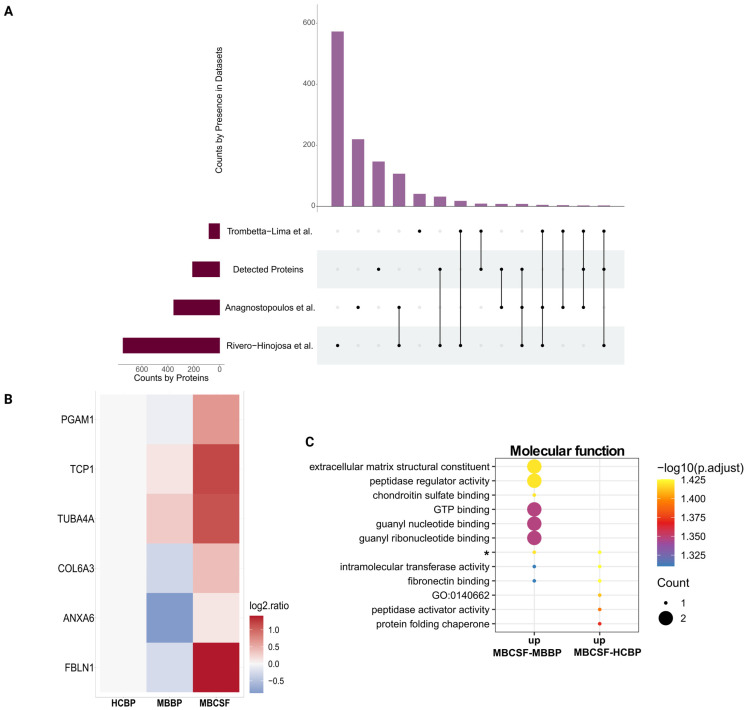
Comparison of proteins detected with our analysis (Detected Proteins) with 3 datasets comprising proteins significantly upregulated in MB-tissue. (**A**) Upset plot illustrating the amount of shared and unique proteins across the 3 selected datasets and Detected Proteins. (**B**) Heatmap presenting the distribution between MBBP and MBCSF and (**C**) Top molecular functions for proteins shared between the 2-Proteins-Cut-Off (at least 2 datasets) and our dataset. * = intramolecular transferase activity, phosphotransferases.

From the final MB protein selection (using the 2-Proteins-Cut-Off), 14 proteins were identified in our dataset (Figure 5A, and Supplementary Figure S20). Out of the six proteins showing differential regulation between the body fluids (Figure 5B), five of them were elevated in CSF. ANXA6 was markedly decreased in MBBP compared to MBCSF, but not HCBP. When the six differentially regulated proteins were subjected to GO analysis, they were linked to ECM-binding functions, including fibronectin binding but not integrin binding (Figure 5C). Notably, the ECM structural constituent was upregulated in MBCSF compared to MBBP, but not HCBP (Figure 5C).

As healthy CSF samples were not included in our study, we used existing protein datasets from non-cancerous CSF (from healthy individuals or patients with congenital hydrocephalus [grades III to V] unrelated to brain tumors). These datasets, and consequently the 2-Proteins-Cut-Off, comprised a large number of proteins (2-Proteins-Cut-Off: 1331 proteins) (Supplementary Figure S22A,B), covering 145 of 210 proteins (69.05%) from our dataset (Supplementary Figure S23A). This overlap prevented identification of a distinct CSF protein signature, and proteins in MBCSF were both up- and down-regulated (Supplementary Figure S23A). On the basis of these findings, we hypothesize that there is not a specific CSF proteome signature and that proteins present in CSF are variable.

Comparing our dataset with Vesiclepedia Top 100 and Exocarta Top 100 (two datasets comprising most commonly listed EV proteins), we found that 14 EV markers were recovered in our study, and they were differently distributed between diseased CSF and BP, but were overall balanced between both fluids (Supplementary Figures S21A,B and S23B). Notably, ITGB1 was listed in both datasets, indicating a central role of integrins in sEVs (Supplementary Figures S21A,B and S23B).

## 3. Discussion

Current standard MB diagnosis relies on invasive tissue biopsies, which limit repeated sampling and, subsequently, monitoring a tumor’s molecular characteristics during disease progression [24]. Liquid biopsy presents a promising alternative, enabling repeated collection of valuable information [25]. Among liquid biopsy analytes, sEVs are particularly valuable as their cargo is protected from degradation, making them suitable for clinical use [4,26]. In addition, sEVs originate from viable cells, potentially providing insights about therapy-resistant cells [4]. However, the lack of standardized sEV isolation protocols impairs progress in this field, as different methods vary in purity and yield [18,19].

In this study, we used UF-SEC to isolate sEVs from the BP and CSF in a pediatric setting. We found that sEV distribution differed between BP and CSF, underlining that the intrinsic characteristics of body fluids influence sEV isolation, even when the same isolation method is employed. Consequently, it is imperative that each sEV preparation method be adapted individually to the body fluid in question.

Our analysis revealed that sEVs in CSF were smaller and consequently, more enriched in later SEC fractions. In contrast, the early fractions, in which sEVs typically elute, were devoid of vesicles. To our knowledge, this observation has not been previously reported [27]. Although BP-sEVs were also detected in these later fractions, the sEV yield was low compared to earlier fractions, prompting us to exclude these fractions for further analysis. This observation raises the question of why CSF contains only smaller sEVs and lacks larger ones.

The size of sEVs is influenced under alia by their biogenesis pathway, protein composition, cell type, and biological context [5,28,29,30]. Interestingly, characterization of sEVs isolated from different cell types showed slight differences in their sizes, suggesting that cell morphological characteristics (cell type) are not a determining factor for sEV size [31]. This would not explain why sEVs from CSF appear smaller than those from BP. One explanation may lie in the immune-privileged environment of the CNS, where minimal immune cell infiltration under physiological conditions leads to low inflammatory activity [32]. Since inflammation can modestly increase sEV size, its absence may promote the release of smaller vesicles [32].

Additionally, the fluid environment also affects EV size [33]. In protein-rich plasma, sEVs form a thick protein corona that surrounds the lipid membrane and can increase their diameter [34]. In contrast, low-protein fluids produce a thinner corona, resulting in smaller measured sizes [34]. For example, plasma sEVs have a dense corona, while sEVs from urine have minimal corona, more accurately reflecting their true size [35]. This difference may explain why CSF-derived sEVs appear smaller than plasma-derived ones.

Sample processing settings, including centrifugation speed and pore sizes of SEC columns and filters, can greatly influence the measured sEV size [36]. However, since we applied identical processing methods to both fluids, these factors do not account for our observed differences.

Faced with free protein contamination, we did not achieve isolation of highly pure sEVs in both body fluids. In CSF, total protein levels measured in the CSF’s diagnostic work-up correlated with free protein concentrations in the pooled fractions, complicating the attribution of specific proteins to sEVs. Since we used the BCA assay for measurement of free protein concentration, without prior lysis, it likely measured only free proteins and loosely bound corona proteins, while tightly bound and integral EV proteins were probably not detected [37]. Consequently, these free proteins are also present in the proteomic data. Protein functions identified by our GO and Cytoscape analyses therefore cannot be exclusively attributed to sEVs, as they may also derive from free, soluble proteins in the sample. This clearly limits our ability to draw definitive conclusions about EV-specific proteins and their functions in MB using our isolation method. Nevertheless, it allows for a comprehensive diagnostic one-stop-shop approach by capturing a broad range of circulating proteins. These findings also emphasize the need to improve methods for enhancing sEV purity.

EV proteins are known to act as key mediators in cancer progression by regulating signaling pathways and participating in various tumor-promoting processes [11,38]. We therefore assessed the informative value of sEV proteins from BP versus CSF for MB, the most common malignant brain tumor in children [1,2].

Subjecting sEV proteins detected by LC-MS/MS to GO analysis, we found ECM organization, its interaction with cells through integrins, and the complement system to be key functions that were differentially regulated between sEVs derived from MBCL, MBBP and MBCSF, and HCBP.

The ECM has gained increasing interest in cancer research. It is now recognized as being shaped by cancer and, in turn, acting as a key regulator of cancer development and progression, the tumor microenvironment (TME), and the formation of pre-metastatic niches [39,40]. CNS tumors have been found to be enriched in ECM proteins [41]. Moreover, ECM proteomic profiles differ between cancerous and healthy tissue, as well as between tumor entities and even MB subgroups [41,42]. However, the structure and interaction of the various ECM components are complex, partly due to their dynamic properties, compromising the understanding of these processes [43]. With our approach, we were able to detect various ECM components that are predominant in the CNS, including fibronectin, chondroitin sulfate proteogylcans, tenascins, and collagens [44].

Collagen is a major ECM and TME component with both pro- and anti-tumor roles [45]. Tumor cells modulate collagen production, which in turn influences their polarity and signaling through various pathways, including via integrins [45]. In cancer, collagen has been found to induce exosome release [46]. We identified several collagens, of which COL5A1 and COL9A1 were significantly enriched in MBCL and MBCSF. COL5A1, whose elevated expression levels have been reported in various cancer entities, including glioblastoma, plays a role in tumor progression, metastasis, and chemo-resistance, and is associated with poor prognosis [47,48,49]. Interestingly, abundance levels of COL4A1, to which similar properties have been attributed, were decreased in MBCSF, while they were elevated in MBCL [50]. Notably, we did not detect elevated collagen VI chains in MBBP and MBCSF, despite prior reports of their abundance in MB tumor tissue [41].

**Figure 6:**
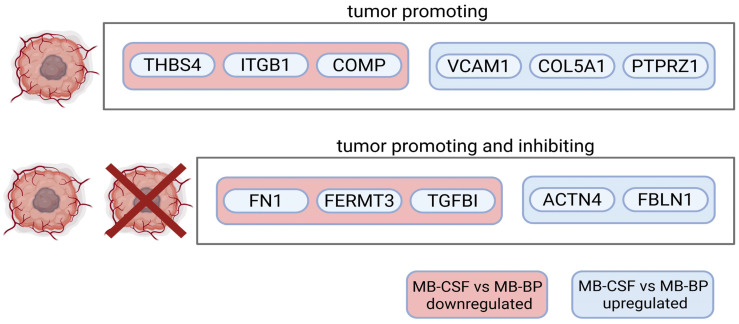
Graphic depiction of proteins with pro- or pro- and anti-tumorigenic potential differentially regulated between MBBP and MBCSF.

Integrins are transmembrane receptors that mediate cell–ECM adhesion and are involved in several processes related to tumor progression [51]. They are also present on EVs, where they have been shown to regulate EV uptake and may drive organ-specific metastasis [52]. Our analysis highlighted integrins, particularly β1 and β3, related adhesion proteins, and signaling pathways as key elements among the top listed GO terms and Cytoscape pathways overall and of proteins differentially regulated across the conditions. Notably, integrin expression and binding profiles varied between MBBP and MBCSF. A PubMed search of the integrin-binding proteins differentially regulated between these two revealed that both conditions contained proteins with pro-tumorigenic functions, as well as proteins with both pro- and anti-tumor potential [47,53,54,55,56,57,58,59,60,61,62] (Figure 6).

The opposing functions (pro- and anti-tumorigenic) observed within a single body fluid may reflect the mixed origin of sEVs, which likely include contributions not only from tumor cells and the tumor microenvironment but also from healthy tissue. As a result, the sEV proteome can carry both pro- and anti-tumor signals. Further research is needed to unravel the complexity of sEV roles and the individual pro- and anti-tumor mechanisms transported in body fluids. Cell-specific sEV isolation could extensively contribute to our understanding of circulating signals and communication.

One of the top Cytoscape pathways across all analyses was “integrins in angiogenesis”. Additionally, angiogenesis was negatively regulated in MBBP compared to HCBP. As a hallmark of cancer, its regulation is highly relevant to tumor biology. Tumor vasculature often disrupts the BBB, forming a BTB that may allow CNS tumor biomarkers to enter the bloodstream. Additionally, Phoenix et al. demonstrated that BBB integrity varies between MB subtypes [63]. They described that a leaky BBB in WNT-MB allows chemotherapy to enter the CNS in high dosages, effectively treating the tumor and improving prognosis [63]. As a leaky barrier could lead to the detection of biomarkers in blood, which were otherwise only detectable in CSF, monitoring the integrity of the BBB/BTB is of great importance for liquid biopsy and requires further research.

The complement system was one of the most enriched terms in our GO and Cytoscape pathway analyses, which could be due to intrinsic characteristics of proteomes from body fluids, as associated proteins were less abundant in MBCL. However, the complement system is also attributed to both pro- and anti-tumorigenic roles in cancer [64].

In sum, we demonstrated the involvement of MBBP in several tumor-associated processes, including integrin binding, angiogenesis and the complement system. However, differences in expression levels of MBBP (compared to HCBP) were too small to be detected by the standard algorithm. Furthermore, MB-proteins were not enriched in MBBP. This finding may also be due to the higher dilution of tumor-derived sEVs in a considerably larger volume of blood (compared to CSF volumes) with a higher concentration of sEVs, which consequently results in a diminished sensitivity.

In contrast, we found that MBCSF, compared to MBBP, recovered functions of proteins enriched in MBCL, including extracellular matrix organization and cell–matrix adhesion.

Consistent with previous work on successful biomarker detection in CSF, we were able to detect MB proteins in our CSF-sEV preparations [16,17,65,66,67]. Consequently, our findings suggest that CSF-sEVs may be more informative than BP-sEVs for MB diagnosis. However, this observation requires further validation in larger cohorts.

In a clinical setting, CSF acquisition is more expense than BP, and CSF is most often not continuously available. Additionally, children, depending on their age, are being sedated for lumbar puncture, and there are considerable risks for side effects caused by lumbar puncture, including postpuncture pain syndrome.

In this study, we optimized a low-volume UF-SEC workflow and found that CSF-derived sEVs are smaller and elute in later SEC fractions than plasma-derived sEVs. Comparative proteomic analysis showed enrichment of medulloblastoma-associated proteins in CSF sEVs, suggesting that CSF-sEVs may be more informative than BP-sEVs for diagnosis and monitoring. A key limitation is the absence of healthy CSF controls, which we mitigated by benchmarking against published healthy CSF proteomes; however, interindividual and methodological heterogeneity in those datasets likely limits the added value of a small in-house healthy CSF set. Additional limitations include the small cohort size and co-isolation of soluble proteins, which temper the strength of our conclusions. Overall, our findings support the need for fluid-specific sEV isolation and standardized workflows, and nominate extracellular matrix components, integrins, and the complement system as candidate pathways in medulloblastoma progression that warrant validation in larger, independent, and longitudinal cohorts across the disease molecular subgroups.

## 4. Materials and Methods

### 4.1. Collection and Processing of Patient Samples

Blood plasma and CSF samples were obtained from patients diagnosed with primary CNS tumors, including MB, in their childhood or adolescence (14 blood-CSF pairs from 11 patients; age range 0.7–20 years; median age 4.35 years at sample collection; 6 females), after oral and written consent was obtained. All patients were admitted to the University Hospital of Essen (Department of Neurosurgery/Department of Pediatrics III). The study was approved by the Ethics Committee of the Medical Faculty of the University Hospital of Duisburg-Essen (19-9095-BO).

For sEV characterization (Figure 1), samples from various pediatric CNS tumor entities were included to demonstrate the broad applicability of the isolation method. These tumor entities comprised MB, sarcoma, glioblastoma, astrocytoma, ependymoma, embryonal tumor with multilayered rosettes (ETMR), and atypical teratoid/rhabdoid tumor (AT/RT) (see Supplementary Table S1 for details). For proteomic profiling and all subsequent analyses, only MB samples (n = 3) were used (classical histology & Group 4, anaplastic histology & WNT, classical histology & WNT). Histological and molecular classification, together with detailed patient and sample characteristics, are provided in Supplementary Table S1. Additionally, blood samples from healthy donors (n = 3) were collected as controls.

Blood was drawn via venous puncture using a 12-gauge needle or collected through a central line, while up to 2 ml of CSF was obtained during lumbar puncture, surgery, or from an extraventricular drain. Samples were stored at 4 °C, then centrifuged at 500× *g* for 10 min at 4 °C and at 3000× *g* for 20 min at 4 °C to remove cells and debris using Rotixa 50 RS centrifuge (Hettich Zentrifugen, Bäch, Switzerland). Processed BP and CSF were stored at −80 °C until further use.

CSF samples with visible blood contamination were excluded from the study.

### 4.2. Isolation of Small Extracellular Vesicles from Blood Plasma and Cerebrospinal Fluid (UF-SEC)

sEVs were isolated using UF-SEC. Processed blood plasma (500 µL) or available CSF volumes were first centrifuged at 1500× *g* for 10 min (Room Temperature [RT]) and then at 10,000× *g* for 10 min (RT) to remove large vesicles, including apoptotic bodies, using the centrifuge 5424R (Eppendorf SE, Hamburg, Germany). Supernatants were adjusted to 2 ml with 0.2 µm-filtered DPBS and loaded onto Amicon^®^ Ultra centrifugal filters (10 kDa, Merck Millipore Ltd., Tullagreen, Ireland). Samples were centrifuged at 4000× *g* (RT) to concentrate them to 500 µL. Plasma samples underwent two additional wash steps with Dulbecco’s Phosphate-Buffered Saline (DPBS) in order to prevent clogging of the filter. Concentrated samples were then subjected to size exclusion chromatography using IZON qEVoriginal (35 nm) columns (Izon Science Ltd., Addington, New Zealand). After voiding with 3 mL DPBS, samples were eluted in 500 µL steps, collecting 10 fractions (F1–F10; total 5 mL), which were then stored at −80 °C until further use.

To compare CSF and blood plasma as well as CSF samples, all results obtained from CSF samples for sEV characterization were normalized to 500 µL using the following formula:

(original CSF volume) × 500 µL/(original CSF volume) = normalized value.(1)

### 4.3. Isolation of Small Extracellular Vesicles from Medulloblastoma Cell Lines

To isolate sEVs from supernatants of the MB cell lines DAOY, ONS76, and UW228, cells were cultivated for 72 h in Dulbecco’s minimal essential medium (DMEM; Gibco^®^ Life Technologies Corp., Paisley, UK) supplemented with 10% EV-depleted fetal bovine serum (Biowest, Nuaillé, France) and 1% penicillin/streptomycin (Gibco^®^ Life Technologies Corp., Paisley, UK). Supernatants were collected, and cells and cell debris were removed by centrifugation. sEVs were then isolated using a combination of tangential flow filtration, size exclusion chromatography, and ultrafiltration as previously described by Chetty et al. [5].

### 4.4. Characterization of Small Extracellular Vesicles

sEV characterization was performed using BP and CSF samples from patients diagnosed with primary CNS tumors in their childhood or adolescence (Figure 1, Supplementary Table S1).

#### 4.4.1. Particle Concentration

The particle concentration was determined by Nanoparticle Tracking Analysis (NTA) as previously described, with adapted sample dilutions for optimal measurement [5]. Briefly, the ZetaView BASIC PMX-120 instrument (Particle Metrix GmbH, Inning am Ammersee, Germany) equipped with NTA 2.0 analysis software was calibrated with 100 nm standard beads before measuring the diluted samples. The following settings were selected: positions—11, cycles—5, minimum size—5 nm, maximum size—150 nm, trace length—15 s, sensitivity—75%, shutter speed—75 ms, and frame rate—30.

#### 4.4.2. Protein Concentration

The free protein concentrations in each individual fraction, as well as in the pooled fractions, were measured using the Pierce (TM) BCA Protein Assay Kit (Thermo Scientific, Waltham, MA, USA) following the manufacturer’s instructions. For optimal measurement, the dilution of BP samples was adapted accordingly. The absorbance at 562 nm was measured using a Tecan Infinite^®^ 200 Pro plate reader (Tecan, Grödig, Austria).

To estimate sEV purity, a particles per protein ratio was calculated using the particle count (determined with NTA) and free protein concentration (determined with BCA).

#### 4.4.3. Bead-Assisted Flow Cytometry

The tetraspanin CD81 is a common surface marker used to characterize isolated sEVs. To screen for the presence of CD81 in our sEV preparations, BP and CSF sEV fractions were semi-quantitatively analyzed by flow cytometry using magnetic beads, as previously described by Chetty et al. [5]. Briefly, 5 µL of aldehyde-sulfate latex beads (4 µM, Invitrogen, Carlsbad, CA, USA) were incubated with 20 µL of sEV sample for 30 min (RT) on a rotating shaker (350 rpm). After adding 200 µL of 0.2 µm filtered DPBS (Millex™ PVDF syringe filter, Merck, Tullagreen, Ireland), samples were incubated again under the same conditions (30 min, 350 rpm, RT). sEV-bead complexes were pelleted with 300 µL DPBS (2000× *g*, 5 min, RT), resuspended in 20 µL of 5% Bovine Serum Albumin (BSA) (Carl Roth, Karlsruhe, Germany) to block non-specific binding, and incubated (30 min, 350 rpm, RT). After washing with 200 µL DPBS and centrifugation (2000× *g*, 5 min, RT), the pellet was stained with 5 µL CD81-FITC antibody (B25329, Beckman Coulter, Marseille, France) for 30 min (RT, 350 rpm) in the dark. Unbound antibody was removed by washing with 700 µL DPBS (2000× *g*, 5 min, RT). The final pellet was resuspended in 1 mL DPBS and transferred to the Fluorescence-activated cell sorting (FACS) tubes (Sarstedt, Nümbrecht, Germany). Data were acquired using the CytoFLEX flow cytometer (Beckmann Coulter, Sangtian Island, Suzhou, China) and analyzed with FlowJo V10 software to determine MFI and CD81-positive bead counts.

The following controls were included in the experiment: beads only (for selection of single beads), beads + antibody (to select the positive population), and beads + BSA ± antibody (to assess BSA blocking efficiency).

#### 4.4.4. Transmission Electron Microscopy (TEM) and Measurement of sEV Size

Negative staining was performed at the Electron Microscopy Unit (EMU) of the Imaging Center Essen (IMCES) as previously described [5]. Fractions with the highest MFI values for CD81 (BP: F2–F6, CSF: F5–F9) were pooled for analysis. A Formvar-carbon-coated 200 mesh copper grid (#S162, PLANO GmbH, Wetzlar, Germany) was exposed to a glow discharging for 30 s at 15 mAmpere (easiGlow [TM], TedPella Inc., Redding, CA, USA) to create a hydrophilic surface. Samples (3 µL) were added on top of the grid and negatively stained with 10 µL of 1.5% aqueous phosphotungstic acid solution (*w*/*v*, 2635.1, Carl Roth, Karlsruhe, Germany) for 1 min. After removing excessive fluid, the grids were dried for at least 5 min under ambient air. Images were generated using a JEOL JEM 1400Plus (JEOL Ltd., Tokyo, Japan) operating at 120 kV and with a 4096 × 4096 pixel CMOS camera (TVIPS, Gauting, Germany). Sixteen-bit images were taken using the image acquisition software EMMENU (version 4.09.83). The diameter of particles with typical EV morphology was measured in pixels using ImageJ (version 1.52a).

### 4.5. Mass Spectrometry and Data Analysis

Sample preparation, liquid chromatography-tandem mass spectrometry (LC-MS/MS), and data processing were performed at the EMBL Proteomics Core Facility (Heidelberg, Germany) as described herein. Initial data analysis was performed at the EMBL Proteomics Core Facility, and subsequent analyses were conducted by our working group using modified code originally written by the EMBL Proteomics Core Facility.

#### 4.5.1. Sample Preparation

Three matched pairs of BP and CSF samples from MB patients, alongside three BP samples from healthy donors and three MB cell lines, were selected for preparation of sEV proteins and further proteomic analysis. Reduction in disulfide bonds on cysteine was performed with dithiothreitol (56 °C, 30 min, 10 mM in 50 mM HEPES, pH 8.5) followed by alkylation with 2-chloroacetamide (room temperature, in the dark, 30 min, 20 mM in 50 mM HEPES, pH 8.5). The SP3 protocol was used for sample clean-up and trypsin (sequencing grade, Promega, Madison, WI, USA) was added in an enzyme to protein ratio 1:50 for overnight digestion at 37 °C (in 50 mM HEPES) [68,69].

Peptides were labelled with TMT16plex Isobaric Label Reagent (Thermofisher, Rockford, IL, USA) according to the manufacturer’s instructions [70]. In brief, of 0.8mg reagent dissolved in 42ul acetonitrile (100%) 4 ul was added and incubated for 1 h room temperature. The reaction was with 4 ul 5% hydroxylamine for 15 min. RT. Samples of a set were combined and desalted on an OASIS^®^ HLB µElution Plate (Waters, Milford, MA, USA).

#### 4.5.2. LC-MS/MS P2258

An UltiMate 3000 RSLCnano LC system (Thermofisher, Rockford, IL, USA) equipped with a trapping cartridge (µ-Precolumn C18 PepMap™ 100, 300 µm i.d. × 5 mm, 5 µm particle size, 100 Å pore size; Thermofisher, Rockford, IL, USA) and an analytical column (nanoEase™ *m*/*z* HSS T3, 75 µm i.d. × 250 mm, 1.8 µm particle size, 100 Å pore size; Waters). Samples were trapped at a constant flow rate of 30 µL/min using 0.05% trifluoroacetic acid (TFA) in water for 6 min. After switching in-line with the analytical column, which was pre-equilibrated with solvent A (3% dimethyl sulfoxide [DMSO], 0.1% formic acid in water), the peptides were eluted at a constant flow rate of 0.3 µL/min using a gradient of increasing solvent B concentration (3% DMSO, 0.1% formic acid in acetonitrile).

Peptides were introduced into an Orbitrap Fusion™ Lumos™ Tribrid™ mass spectrometer (Thermofisher, Rockford, IL, USA) via a Pico-Tip emitter (360 µm OD × 20 µm ID; 10 µm tip, CoAnn Technologies, Richland, WA, USA) using an applied spray voltage of 2.2 kV. The capillary temperature was maintained at 275 °C. Full MS scans were acquired in profile mode over an *m*/*z* range of 375–1650, with a resolution of 120,000 at *m*/*z* 200 in the Orbitrap. The maximum injection time was set to 50 ms, and the AGC target limit was set to ‘standard’. The instrument was operated in data-dependent acquisition (DDA) mode, with MS/MS scans acquired in the Orbitrap at a resolution of 30,000. The maximum injection time was set to 110 ms, with an AGC target of 200%. Fragmentation was performed using higher-energy collisional dissociation (HCD) with a normalized collision energy of 34%, and MS2 spectra were acquired in profile mode. The quadrupole isolation window was set to 0.7 *m*/*z*, and dynamic exclusion was enabled with a duration of 20 s. Only precursor ions with charge states 2–7 were selected for fragmentation.

#### 4.5.3. Data Processing, MaxQuant

The raw mass spectrometry data were processed using MaxQuant (v1.6.3.4) [71].

IsobarQuant with Mascot (v2.2.07) was used to process the acquired data, which was searched against a Homo sapiens proteome database (UP000005640, May 2016, 92, 507 entries) plus common contaminants and reversed sequences [72]. The following modifications were included in the search parameters: Carbamidomethyl on Cysteine and TMT16 on lysine as fixed modifications, protein N-term acetylation, oxidation on methionine and TMT16 on N-termini as variable modifications. For precursor ions a mass error tolerance of 10 ppm was used and for fragment ions 0.02 Da was set. Trypsin was set as protease with a maximum of two missed cleavages. The minimum peptide length was set to seven amino acids. At least two unique peptides were required for protein identification. The false discovery rate on peptide and protein level was set to 0.01.

#### 4.5.4. Data Analysis

For the proteomics data analysis, the raw output files of IsobarQuant (protein.txt files) were processed using the R programming environment (ISBN 3-900051-07-0). Initial data processing included filtering out contaminants and reverse proteins. Only proteins quantified with at least 2 unique peptides (with qupm ≥ 2) were considered for further analysis. 210 proteins passed the quality control filters. In order to correct for technical variability, batch effects were removed using the ‘removeBatchEffect’ function of the limma package on the log2 transformed raw TMT reporter ion intensities (‘signal_sum’ columns) [73]. Subsequently, normalization was performed using the ‘normalizeVSN’ function of the limma package (VSN-variance stabilization normalization-[74]). Differential expression analysis was performed using the moderated *t*-test provided by the limma package [73]. The model accounted for replicate information by including it as a factor in the design matrix passed to the ‘lmFit’ function. Proteins were annotated as hits if they had a false discovery rate (FDR) below 0.05 and an absolute fold change greater than 2. Proteins were considered candidates if they had an FDR below 0.2 and an absolute fold change greater than 1.5. Clustering with all hit and candidate proteins based on the median protein abundances normalized by median of control condition was conducted to identify groups of protein similar patterns across conditions. The ‘kmeans’ method was employed, using Euclidean distance as the distance metric and ‘ward.D2’ linkage for hierarchical clustering. The optimal number of clusters (10) was determined using the Elbow method, which identifies the point where the within-group sum of squares stabilizes. GO enrichment analysis was performed using the ‘compareCluster’ function of the ‘clusterProfiler’ package [75], which assesses over-representation of GO terms in the dataset relative to the background gene set. Enrichment was conducted for the following GO categories: Cellular Component (CC), Molecular Function (MF), and Biological Process (BP). The analysis was performed using ‘org.Hs.eg.db’ as the reference database. The odds ratio (‘odds_ratio’) for each GO term was calculated by comparing the proportion of genes associated with that term in the dataset (‘GeneRatio’) to the proportion in the background set (‘BgRatio’). An odds ratio greater than 1 indicates that the GO term is enriched in the dataset compared to the expected background.

The mass spectrometry proteomics data have been deposited to the ProteomeXchange Consortium via the PRIDE partner repository with the dataset identifier PXD067299 [76].

### 4.6. Selection of MB, CSF and sEV Datasets and Comparison of Identified Proteins

ProteomeXchanger Consortium and PubMed were searched for publicly available datasets of CSF and MB proteomes. For MB tissue, we selected datasets from Anagnostopoulos et al., Rivero-Hinojosa et al. (PXD008750), and Trombetta-Lima et al. [41,77,78]. Proteins differentially regulated in MB tissue compared to healthy cerebellar tissue were included in the analysis. For CSF, we used datasets from Lilley et al., Bruschi et al. (2021), and Bruschi et al. (2022) (PXD035292) [79,80,81]. These datasets included CSF controls from healthy individuals or patients with congenital hydrocephalus (unrelated to brain tumors) (grades III to V). All proteins detected in the CSF controls were used for the analysis. For EV proteins, we selected the Top 100 EV markers from Exocarta (http://exocarta.org/exosome_markers accessed on 29 May 2025) and Vesiclepedia (http://microvesicles.org/extracellular_vesicle_markers accessed on 29 May 2025). If the gene names of the proteins were not provided in the dataset, gene names were generated by ID mapping (https://www.uniprot.org/id-mapping accessed on 27 October 2024).

Each dataset was compared to the proteins detected in the LC-MS/MS analysis, and all datasets of one group (CSF, EV, or MB) were compared based on shared proteins. A cut-off was established by selecting proteins detected in at least two datasets (“2-Proteins-Cut-Off”) to neglect non-specific proteins. MB proteins shared between the 2-Proteins-Cut-Off and our dataset were subjected to GO analysis. All bioinformatic analyses were performed with R Studio (software version 4.3.3).

### 4.7. Statistical Analysis

Data were statistically analyzed using GraphPad Prism 10.4.1 (GraphPad Software, Boston, MA, USA), and bioinformatic analyses of proteins were performed using R Studio (software version 4.3.3). Protein correlation was assessed using a two-tailed, non-parametric Spearman’s rank correlation coefficient analysis. A two-tailed Mann–Whitney test was used for sEV size analysis of BP- vs. CSF-sEVs. All results represent independent experiments performed at least three times. The data in the figures are expressed as the mean ± standard deviation.

Data that were found to be statistically significant were represented in the graphs as * for *p* < 0.1, ** for *p* < 0.01, *** for *p* < 0.001, and **** for *p* < 0.0001. Alpha error = 0.05.

## 5. Conclusions

In our study, we successfully adapted sEV isolation by UF-SEC to two different body fluids: blood plasma and CSF. This adaption served as a basis for comparing both fluids regarding their diagnostic potential and the information they provide about tumor’s behavior. We emphasized the importance of tailoring sEV isolation methods to specific body fluids, as sEVs from CSF were smaller in size and eluted in later SEC fractions compared to those from BP. Furthermore, we highlighted the relevance of the ECM and integrins in MB tumor progression and their interaction with the tumor microenvironment. Despite the fact that we provide evidence that BP is affected by the tumor, we suggest that CSF might be superior in terms of diagnostic potential. These findings should be validated in a larger cohort to assess the specific contribution of both body fluids in predicting recurrence, metastasis, outcome, and prognosis. To our knowledge, this is the first comprehensive comparison demonstrating that CSF-sEVs outperform BP-sEVs in providing brain-tumor-related information.

## Data Availability

The original contributions presented in this study are included in the article/Supplementary Material. Additionally, the mass spectrometry proteomics data have been deposited to the ProteomeXchange Consortium via the partner repository with the dataset identifier PXD067299 [66]. Further inquiries can be directed to the corresponding author.

## Supplementary Materials

The following supporting information can be downloaded at: https://www.mdpi.com/article/10.3390/ijms26199279/s1.

## Abbreviations

The following abbreviations are used in this manuscript:

EV	Extracellular vesicle
sEV	Small extracellular vesicle
CSF	Cerebrospinal fluid
BP	Blood plasma
CNS	Central nervous system
MB	Medulloblastoma
UF-SEC	Ultrafiltration–size exclusion chromatography
GO	Gene Ontology
ECM	Extracellular matrix
cfDNA	Cell-free DNA
BBB	Blood–brain barrier
RT	Room temperature
DPBS	Dulbecco’s Phosphate-Buffered Saline
NTA	Nanoparticle Tracking Analysis
BCA	Bicinchoninic acid assay
BSA	Bovine Serum Albumine
FITC	Fluorescein-isothiocyanat
FACS	Fluorescence-activated cell sorting
MFI	Mean fluorescence intensity
LC-MS/MS	Liquid Chromatography–Tandem Mass Spectrometry
FDR	False discovery rate
MBBP	Blood plasma from medulloblastoma patient(s)
MBCSF	Cerebrospinal fluid from medulloblastoma patient(s)
HCBP	Blood plasma from healthy control(s)
MBCL	Medulloblastoma cell lines
TGOLN2	Trans-golgi network protein 2
FC	Fold change
ANXA6	Annexin A6
ITGB1	Integrin β-1
TME	Tumor microenvironment
COL5A1	Collagen type V alpha 1 chain
COL9A1	Collagen type IX alpha 1 chain
COL4A1	Collagen type IV alpha 1 chain
BTB	Brain-tumor-barrier
WNT	Wingless-related integration site
