# Supplementary material for: Cerebrospinal Fluid-Derived Small Extracellular Vesicles May Better Reflect Medulloblastoma Proteomes than Those from Blood Plasma
Source: Int J Mol Sci. 2025 Sep 23;26(19):9279. doi: 10.3390/ijms26199279 (PMC12524324; doi:10.3390/ijms26199279)
Supplement: Supplementary file 1 [file ijms-26-09279-s001.zip › Supplementary Figure and table titles.pdf]

Supplementary Table S1. Detailed patient and sample characteristics. **Supplementary Table S2.** Full dataset proteomics with medulloblastoma cell lines (DAOY, ONS76 and UW228) data. **Supplementary Table S3.** Cytoscape pathway enrichment analyses. **Supplementary Table S4.** Full dataset proteomics without medulloblastoma cell lines (DAOY, ONS76 and UW228) data.

Figure S1. Distribution of particles and proteins throughout the UF-SEC fractions from BP and CSF. (**A**) Particle concentration (per ml) per fraction of particles from (a) BP and (b) CSF. (**B**) Protein concentration (µg / ml) of soluble, co-isolated proteins per fraction from (a) BP and (b) CSF. (**C**) Correlation analysis of protein concentrations between (a) whole protein concentration from BP and CSF and (b) soluble, co-isolated proteins in BP-sEV and CSF-sEV fractions.

Figure S2. Unclustered heatmap of 210 sEV-proteins detected by LC-MS/MS. * = hCG_2039566 | A0A0U1RRH7 | HIST1H2AB | HIST1H2AG | H2AFX | HIST1H2AD | HIST2H2AC | HIST2H2AA3 | HIST3H2A | HIST1H2AC | HIST1H2AH | HIST1H2AA | HIST1H2AJ | H2AFJ.

Figure S3. K-means clustering of 210 proteins detected in our analysis. (**A**) Top 10 biological processes associated with proteins of each cluster. (**B**) Cnet plot of proteins and associated top 5 biological processes. Note: no association with biological processes were found for proteins from cluster 6. * = adaptive immune response based on somatic recombination of immune receptors built from immunoglobulin superfamily domains.

Figure S4. K-means clustering of 210 proteins detected in our analysis. (**A**) Top 10 cellular compartments associated with proteins of each cluster. (**B**) Cnet plot of proteins and associated top 5 cellular compartments. Note: no association with cellular compartments were found for proteins from cluster 2.

Figure S5. K-means clustering of 210 proteins detected in our analysis. (**A**) Top 10 molecular functions associated with proteins of each cluster. (**B**) Cnet plot of proteins and associated top 5 molecular functions.

Figure S6. Depiction of differentially regulated proteins between MBCL, MBCSF, MBBP and HCBP. (**A**) Top 10 biological processes associated with proteins differentially regulated between two respective conditions. (**B**) Cnet plot of proteins and associated top 5 biological processes. * = adaptive immune response based on somatic recombination of immune receptors built from immunoglobulin superfamily domains.

Figure S7. Depiction of differentially regulated proteins between MBCL, MBCSF, MBBP and HCBP. (**A**) Top 10 cellular compartments associated with proteins differentially regulated between two respective conditions. (**B**) Cnet plot of proteins and associated top 5 cellular compartments.

Figure S8. Depiction of differentially regulated proteins between MBCL, MBCSF, MBBP and HCBP. (**A**) Top 10 biological processes associated with proteins differentially regulated between two respective conditions. (**B**) Cnet plot of proteins and associated top 5 molecular functions. * = extracellular matrix structural constituent conferring tensile strength.

Figure S9. Top 10 GO terms of k-means cluster 2 & 7, 3 & 4, and 9. (**A**) Top 10 (a) cellular compartments enriched in cluster 7 and (b) molecular functions associated with proteins from cluster 2 & 7. Note: no enrichment for cellular compartments was found in cluster 2. (**B**) Top 10 (a) cellular compartments and (b) molecular functions associated with proteins from cluster 3 & 4. (**C**) Top 10 (a) cellular compartments and (b) biological processes associated with proteins from cluster 9.

Figure S10. Gene Ontology enrichment analysis for k-means cluster 1 and 5 & 10. (**A**) Top 10 (a) biological processes, (b) cellular compartments and (c) molecular functions associated with proteins from cluster 1. (**B**) Top 10 (a) biological processes, (b) cellular compartments and (c) molecular functions associated with proteins from cluster 5 & 10.

Figure S11. Gene Ontology enrichment analysis for k-means cluster 6 and 8. (**A**) Top 10 (a) cellular compartments and (b) molecular functions associated with proteins from cluster 6. Note: no enrichment for biological processes was found in cluster 6. (**B**) Top 10 (a) biological processes, (b) cellular compartments and (c) molecular functions associated with proteins from cluster 8. * = extracellular matrix structural constituent conferring compression resistance.

Figure S12. Cnet plot of differentially regulated proteins and associated top 5 (**A**) molecular functions of cluster 2 & 7 and (**B**) biological processes of cluster 3 & 4. * = adaptive immune response based on somatic recombination of immune receptors built from immunoglobulin superfamily domains.

Figure S13. Cnet plot of differentially regulated proteins and associated top 5 biological processes of cluster 8.

Figure S14. Cnet plot of differentially regulated proteins and associated top 5 (**A**) biological processes and (**B**) molecular functions of cluster 9.

Figure S15. Top 10 (a) biological processes and (b) cellular compartments of proteins differentially regulated between (**A**) MBBP and MBCL, (**B**) MBCSF and MBCL, and (**C**) MBBP and MBCSF. * = adaptive immune response based on somatic recombination of immune receptors built from immunoglobulin superfamily domains.

Figure S16. Top 10 (a) biological processes, (b) cellular compartments, and (c) molecular functions of proteins differentially regulated between (**A**) MBCL and HCBP, (**B**) MBCSF and HCBP. * = adaptive immune response based on somatic recombination of immune receptors built from immunoglobulin superfamily domains, ** = extracellular matrix structural constituent conferring tensile strength.

Figure S17. Cnet plot of proteins differentially regulated between MBBP and MBCSF and associated top 5 molecular functions.

Figure S18. Analysis of differential regulation of proteins between MBBP and HCBP based on fold changes. (**A**) Top 10 biological processes for proteins differentially regulated between MBBP and HCBP. (**B**) Cnet plot of differentially regulated proteins and associated top 5 biological processes.

Figure S19. Analysis of differential regulation of proteins between MBBP and HCBP based on fold changes. (**A**) Top 10 cellular compartments for proteins differentially regulated between MBBP and HCBP. (**B**) Cnet plot of differentially regulated proteins and associated top 5 cellular compartments.

Figure S20. Comparison of proteins significantly regulated in MB from 3 selected datasets of MB-tissue with proteins detected in our analysis (Detected Proteins). (A) VennDiagrams depicting the ratio of unique and shared proteins between Detected Proteins and the respective MB-dataset, or the 2-Protein-Cut-Off (comprises proteins differentially regulated between MB tissue and healthy cerebellar tissue in at least 2 of 3 MB-datasets). (B) Cnet plot illustrating proteins shared between the 2-Protein-Cut-Off and Detected Proteins, and associated top 5 molecular functions.

Figure S21. Comparison of common EV-proteins listed in Exocarta (Exocarta Top 100 Exosome Markers) and Vesiclepedia (Vesiclepedia Top 100 EV proteins) with proteins detected in our analysis (Detected Proteins). (**A**) Upset plot illustrating the amount of shared and unique proteins across the 2 selected datasets and Detected Proteins. (**B**) VennDiagrams depicting the ratio of unique and shared proteins between Detected Proteins and the respective EV-protein-dataset, or the 2-Protein-Cut-Off (comprises proteins listed in both datasets).

Figure S22. Comparison of proteins present in CSF from 3 selected datasets with proteins detected in our analysis (Detected Proteins). (**A**) Upset plot illustrating the amount of shared and unique proteins across the three selected

datasets and Detected Proteins. (**B**) VennDiagrams depicting the ratio of unique and shared proteins between Detected Proteins and the respective CSF-protein dataset, or the 2-Protein-Cut-Off (comprises proteins listed in at least 2 of 3 CSF-protein datasets).

Figure S23. Heatmaps depicting proteins shared between Detected Proteins and the 2-Protein-Cut-Off from (A) EV-protein datasets or (B) CSF-protein datasets.
