# Supplementary figures and images for: Cerebrospinal Fluid-Derived Small Extracellular Vesicles May Better Reflect Medulloblastoma Proteomes than Those from Blood Plasma
Source: Int J Mol Sci. 2025 Sep 23;26(19):9279. doi: 10.3390/ijms26199279 (PMC12524324; doi:10.3390/ijms26199279)

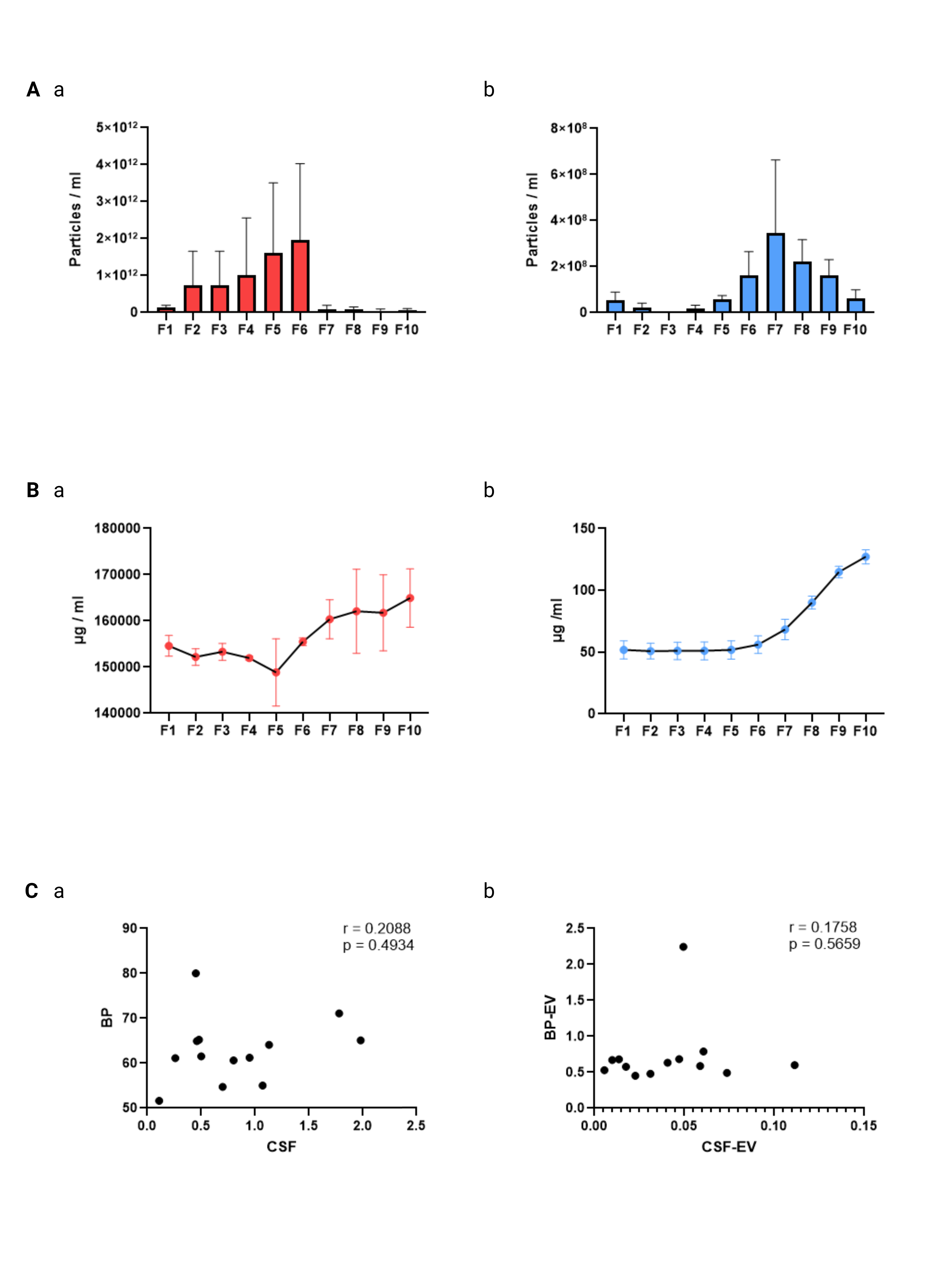

Supplement: Supplementary file 1 [file ijms-26-09279-s001.zip › 1 - Figure S1.png]

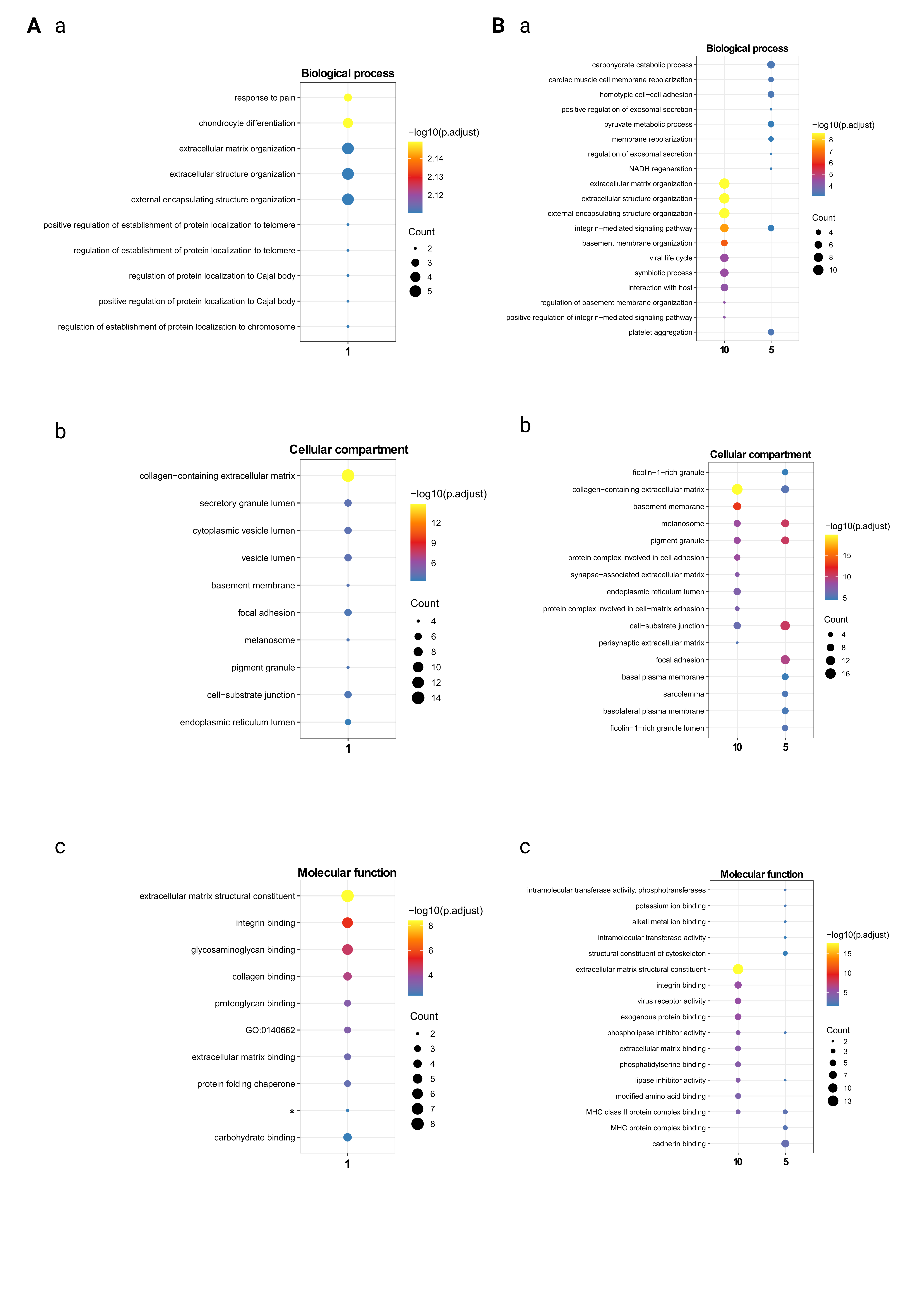

Supplement: Supplementary file 1 [file ijms-26-09279-s001.zip › 10 - Figure S10.png]

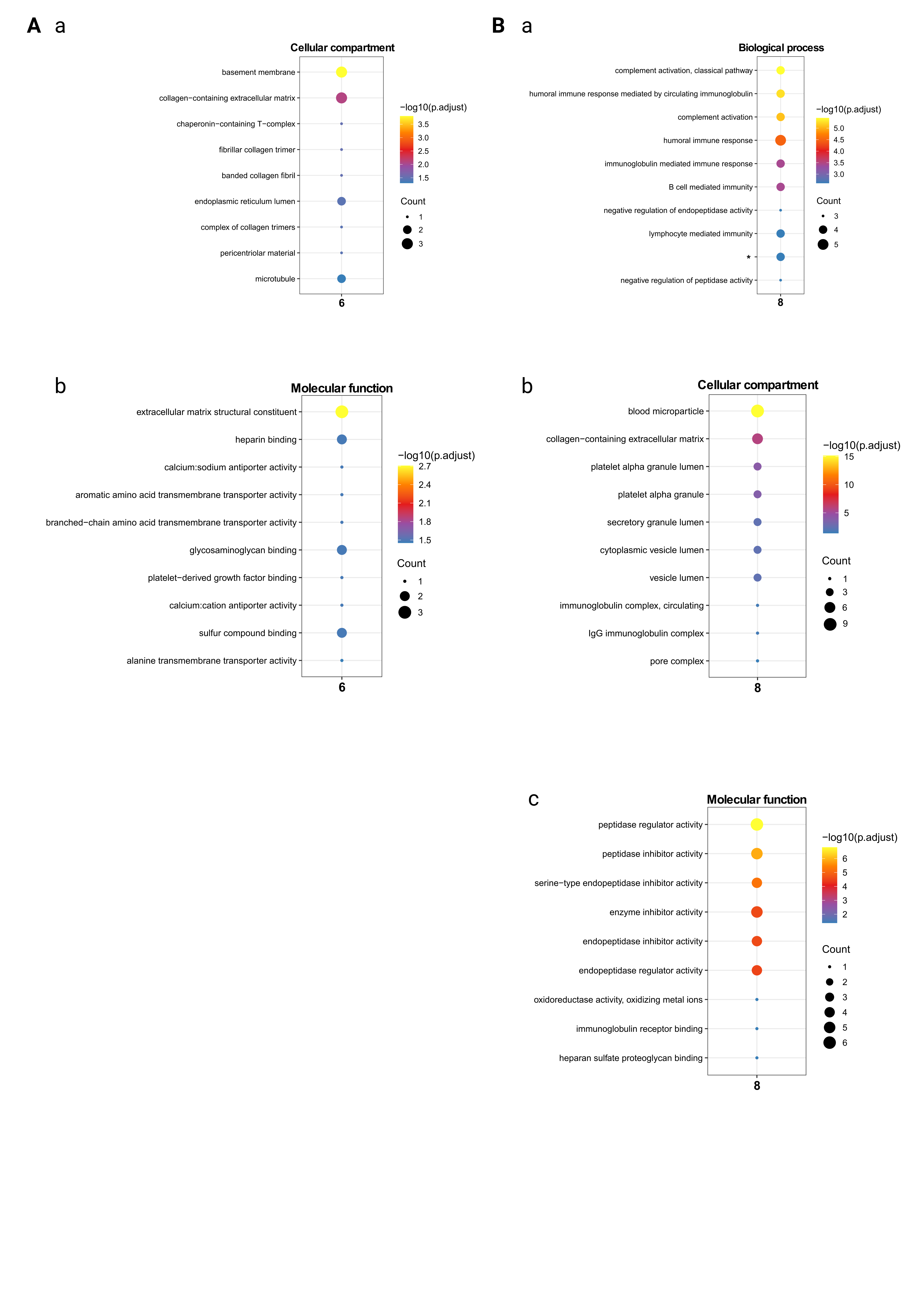

Supplement: Supplementary file 1 [file ijms-26-09279-s001.zip › 11 - Figure S11.png]

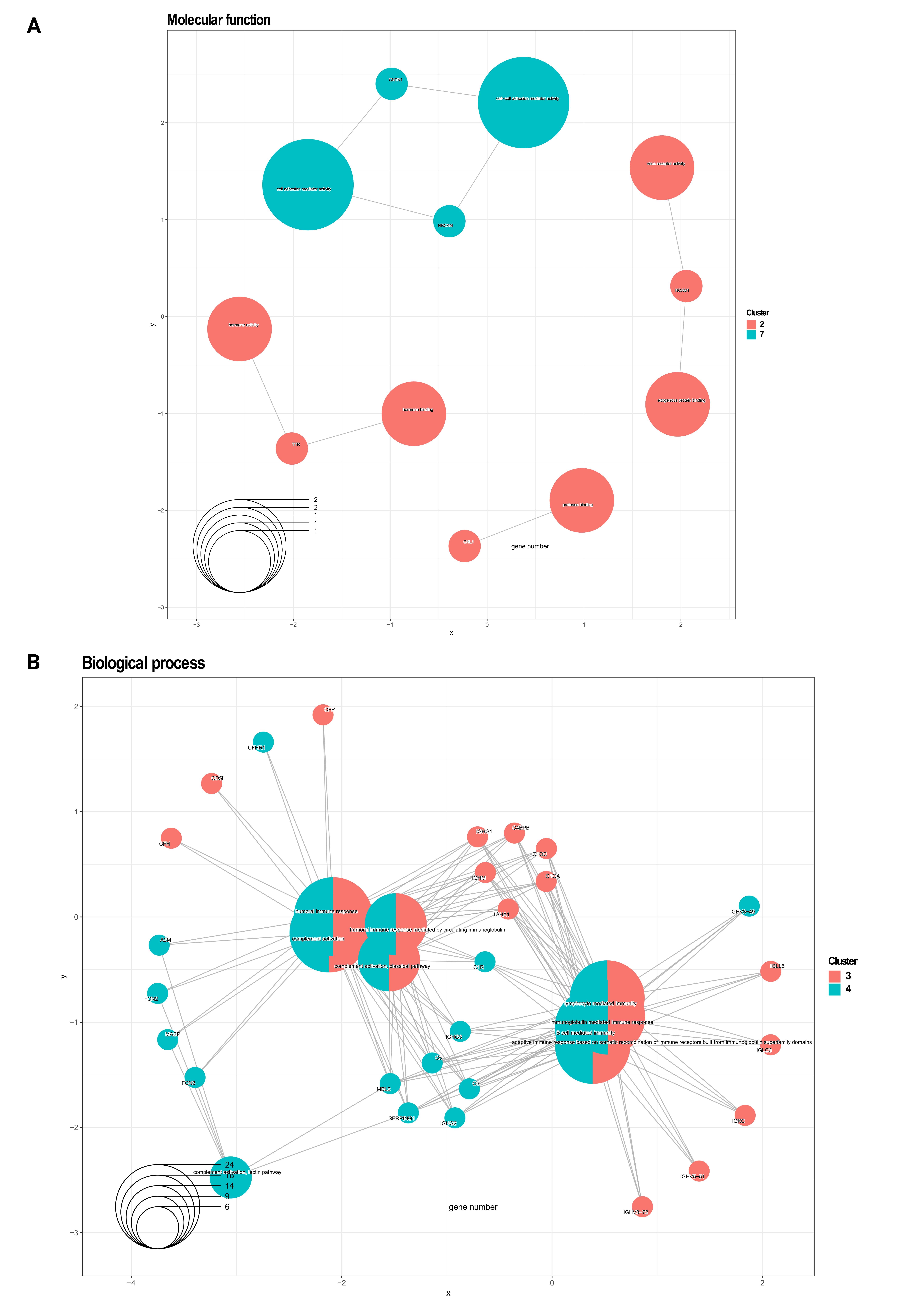

Supplement: Supplementary file 1 [file ijms-26-09279-s001.zip › 12 - Figure S12.png]

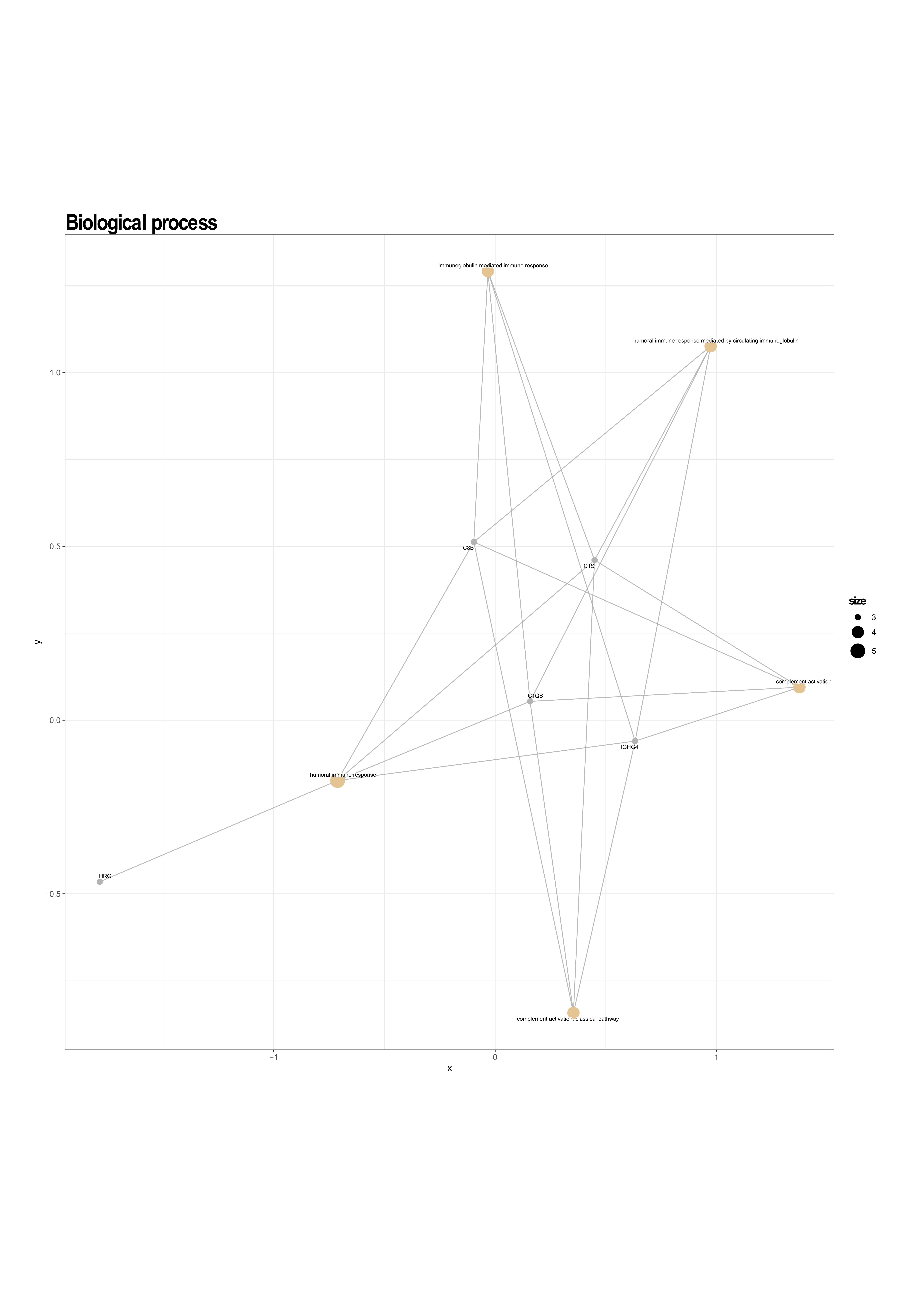

Supplement: Supplementary file 1 [file ijms-26-09279-s001.zip › 13 - Figure S13.png]

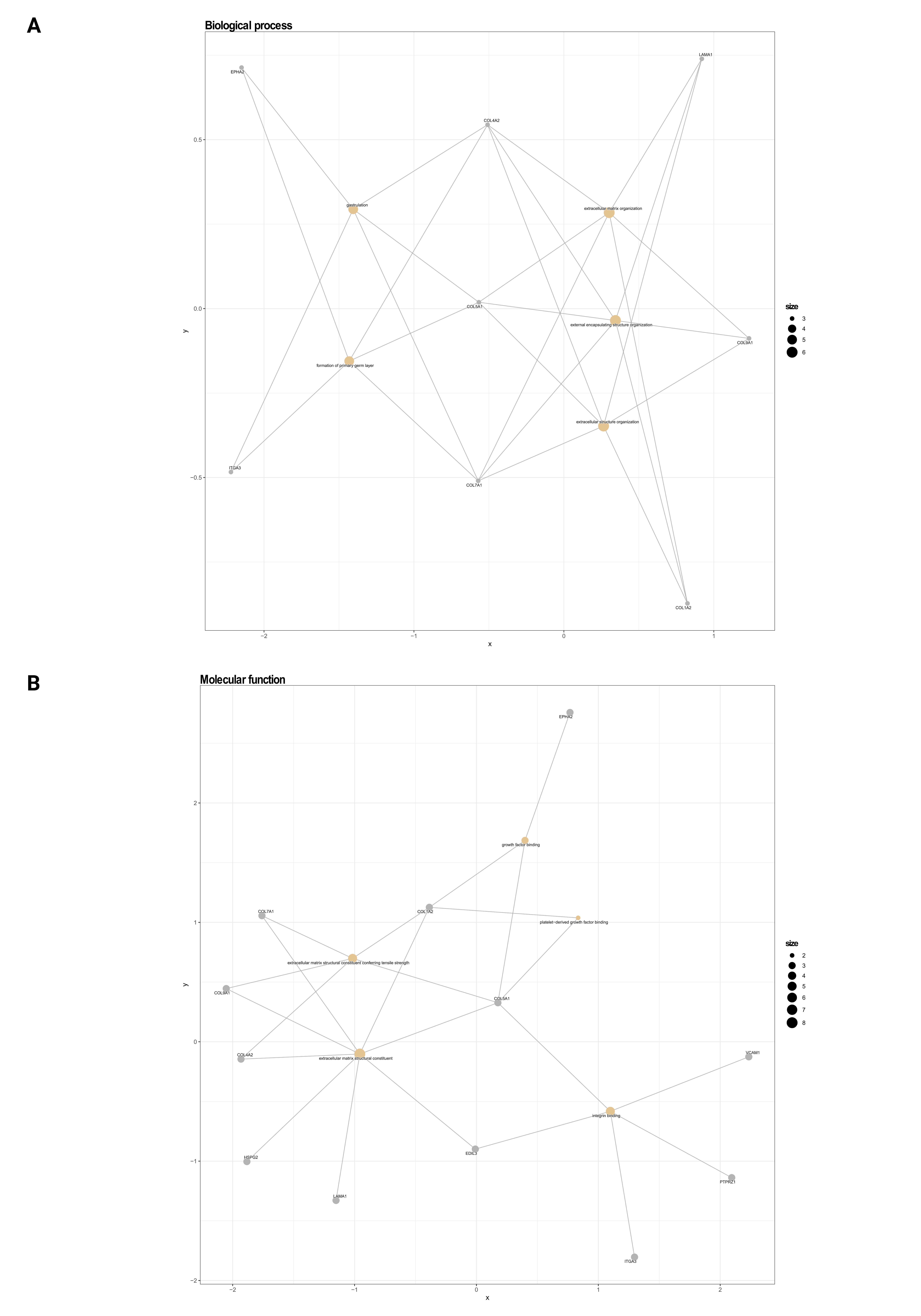

Supplement: Supplementary file 1 [file ijms-26-09279-s001.zip › 14 - Figure S14.png]

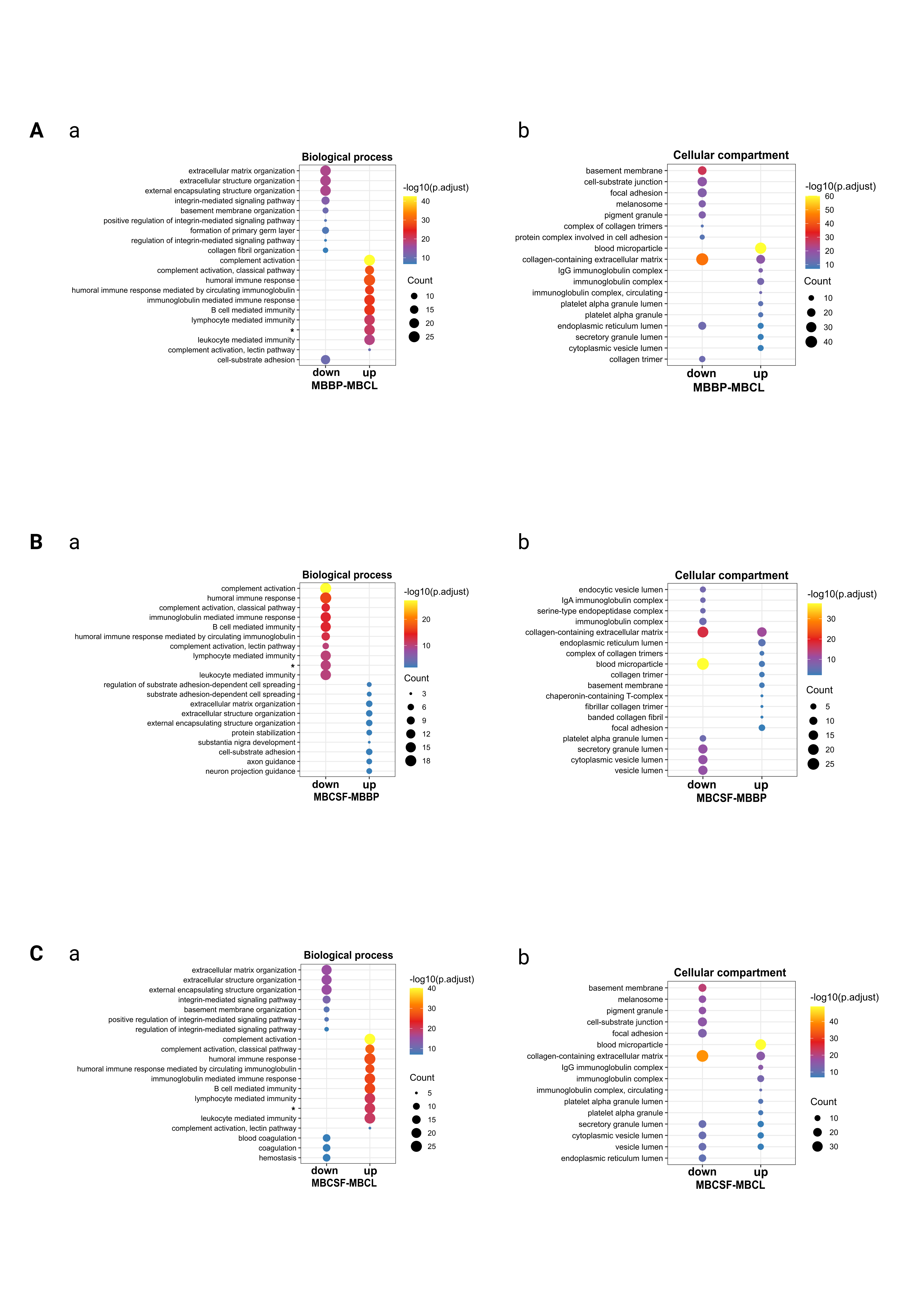

Supplement: Supplementary file 1 [file ijms-26-09279-s001.zip › 15 - Figure S15.png]

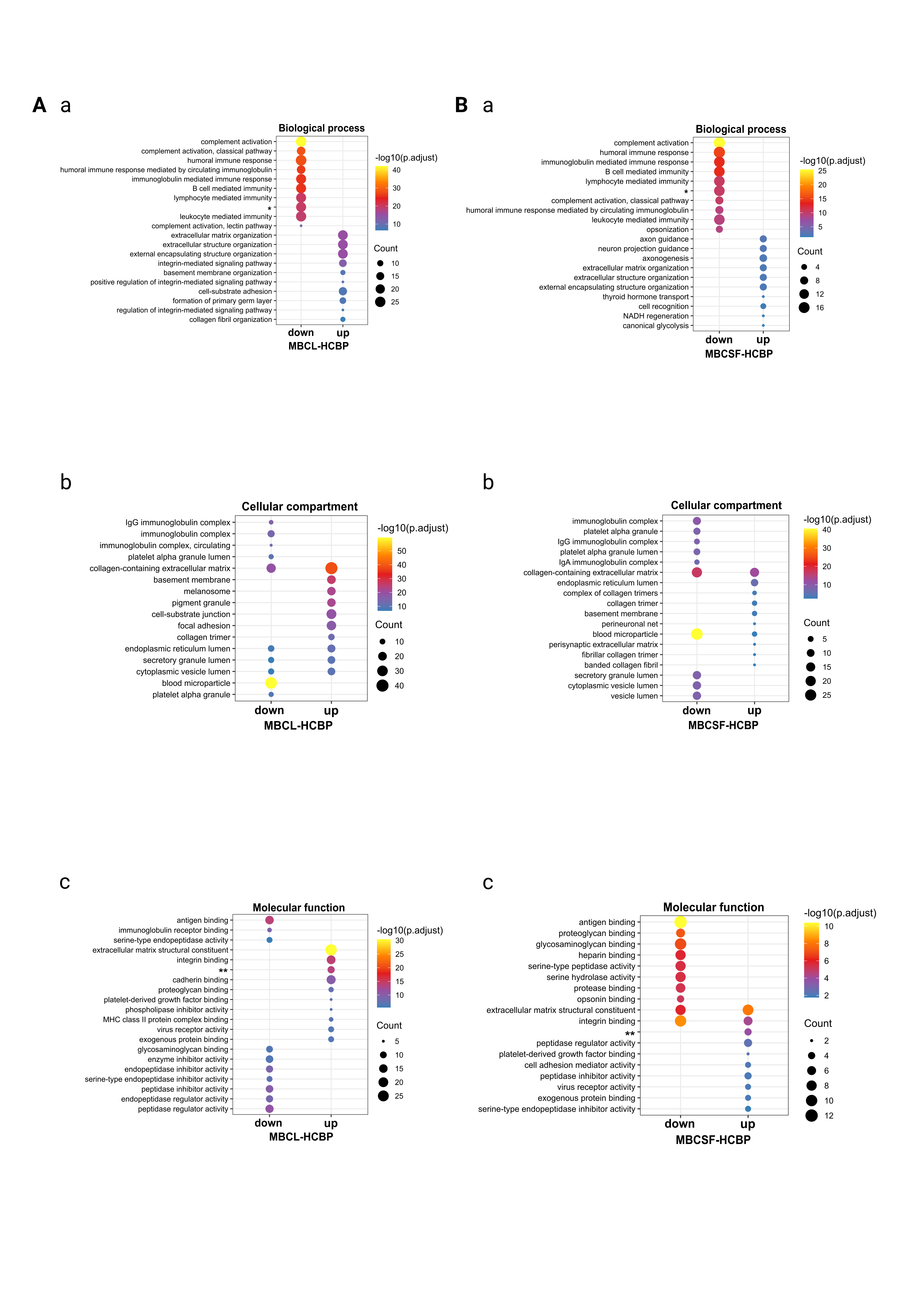

Supplement: Supplementary file 1 [file ijms-26-09279-s001.zip › 16 - Figure S16.png]

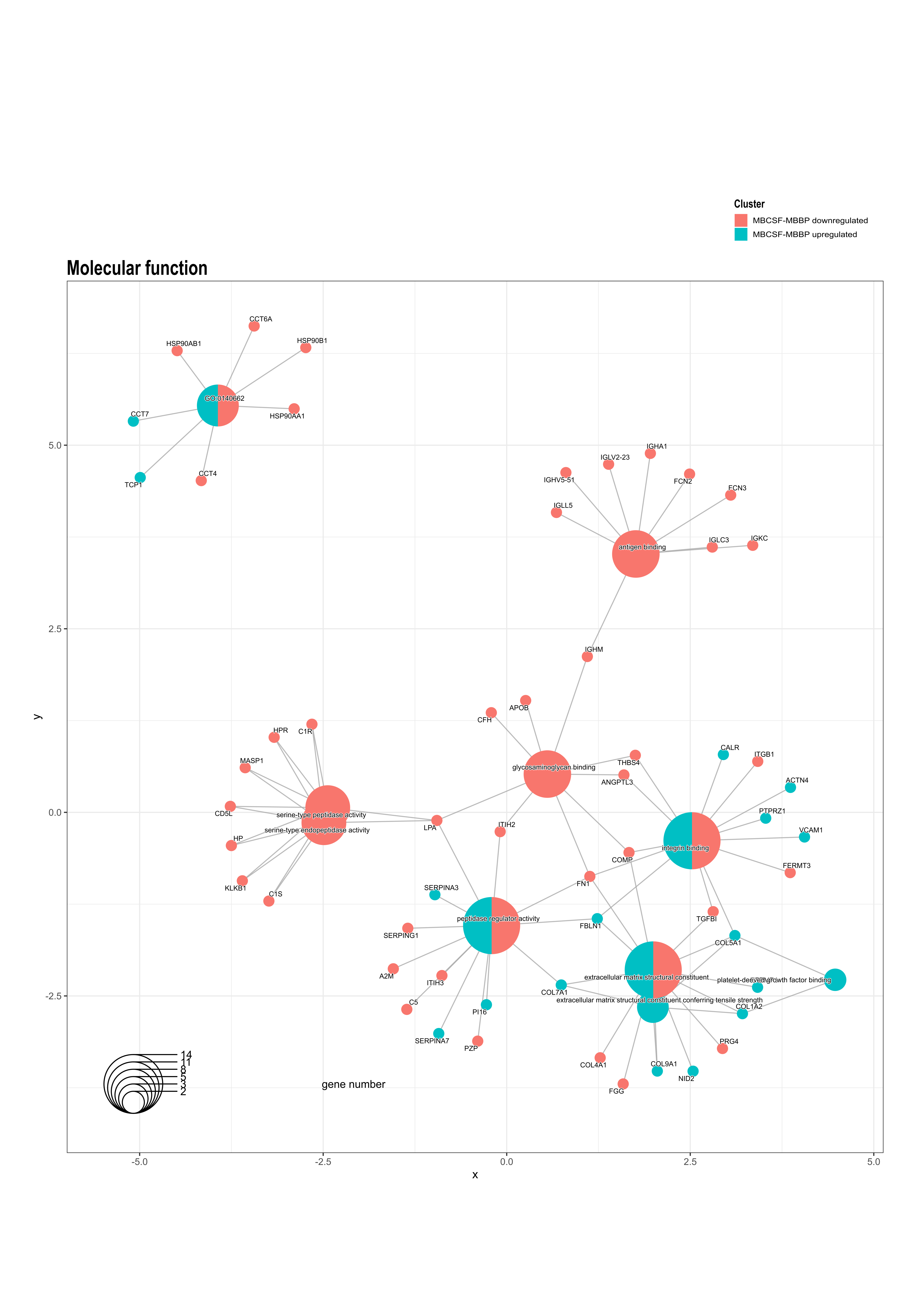

Supplement: Supplementary file 1 [file ijms-26-09279-s001.zip › 17 - Figure S17.png]

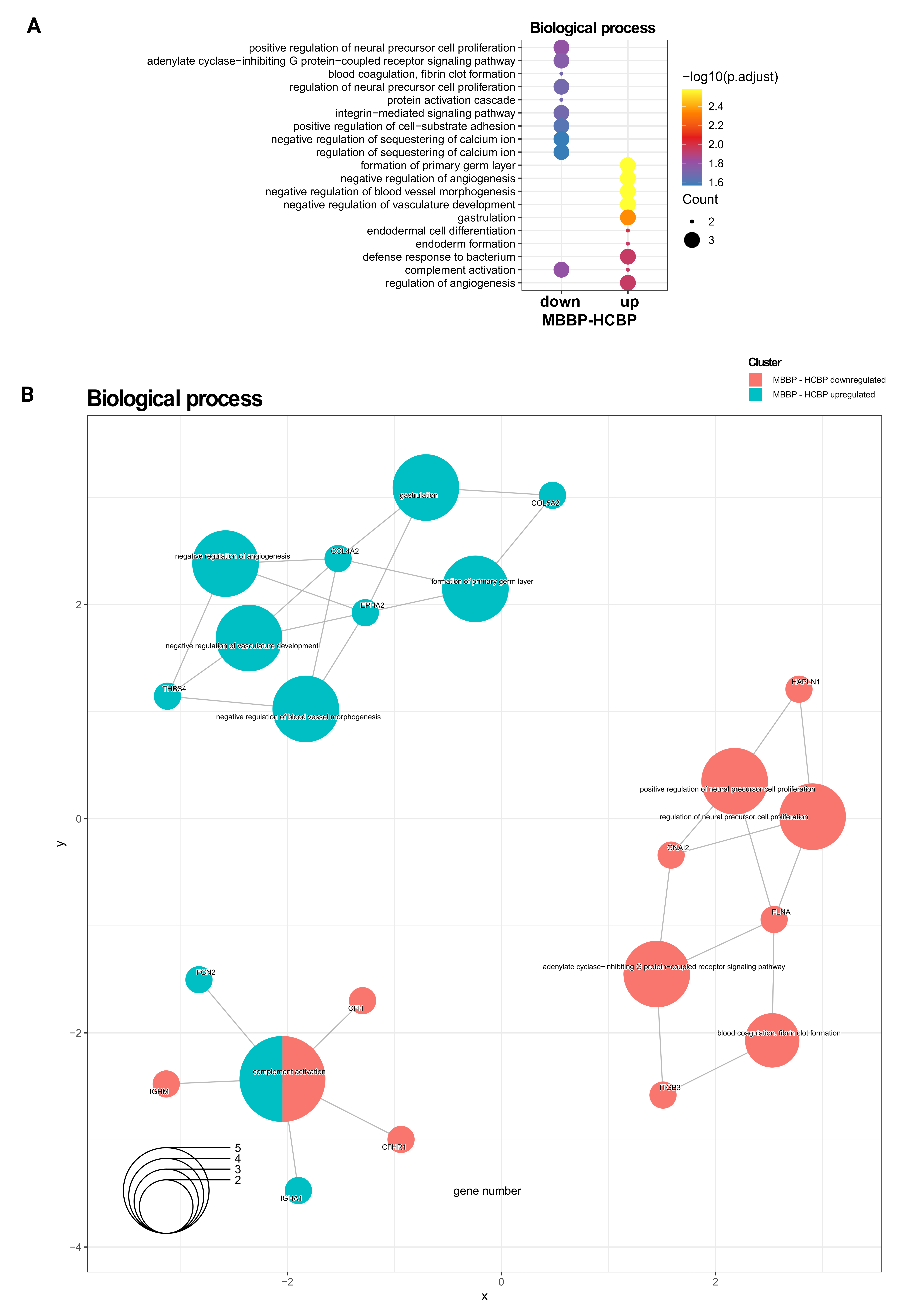

Supplement: Supplementary file 1 [file ijms-26-09279-s001.zip › 18 - Figure S18.png]

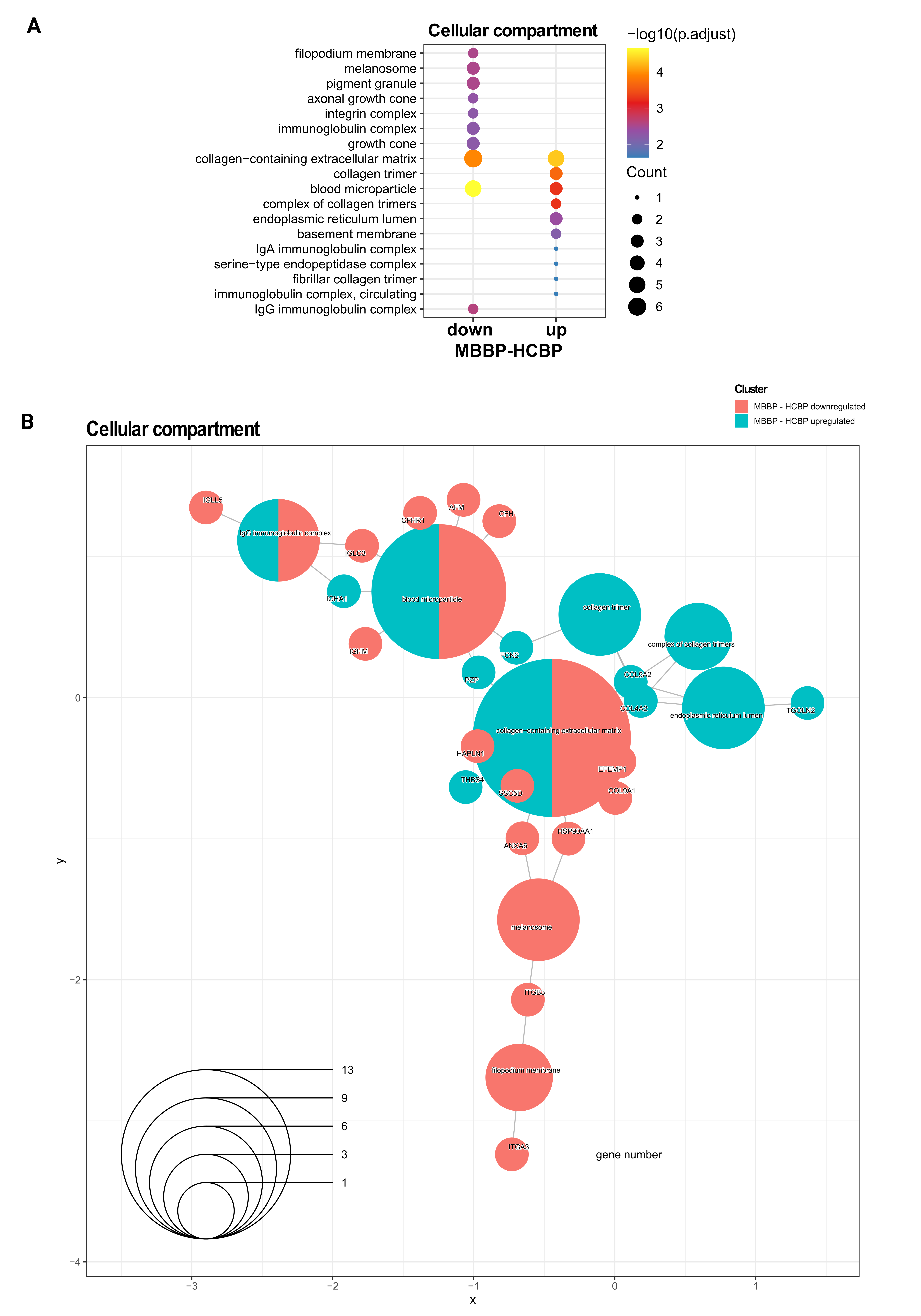

Supplement: Supplementary file 1 [file ijms-26-09279-s001.zip › 19 - Figure S19.png]

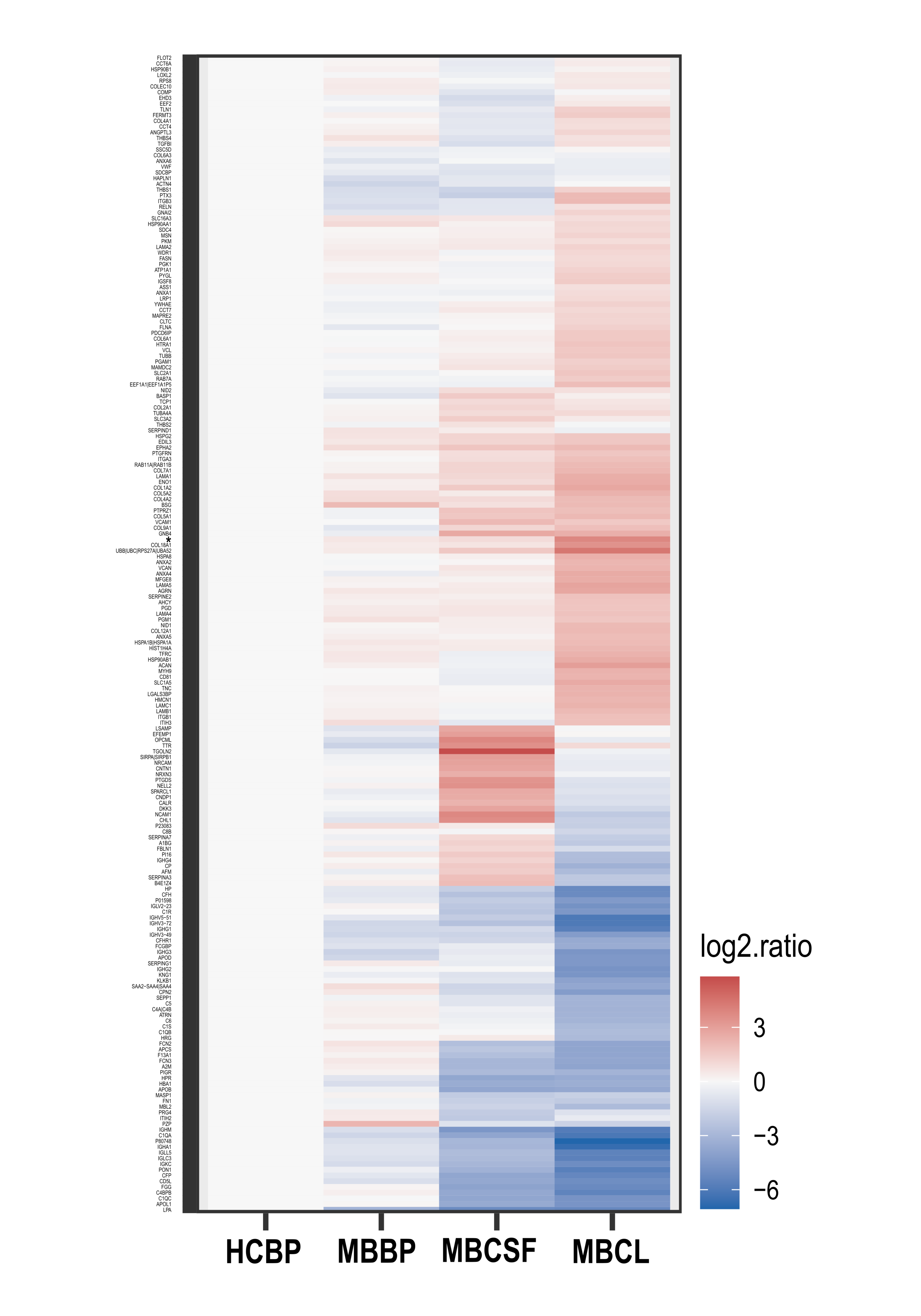

Supplement: Supplementary file 1 [file ijms-26-09279-s001.zip › 2 - Figure S2.png]

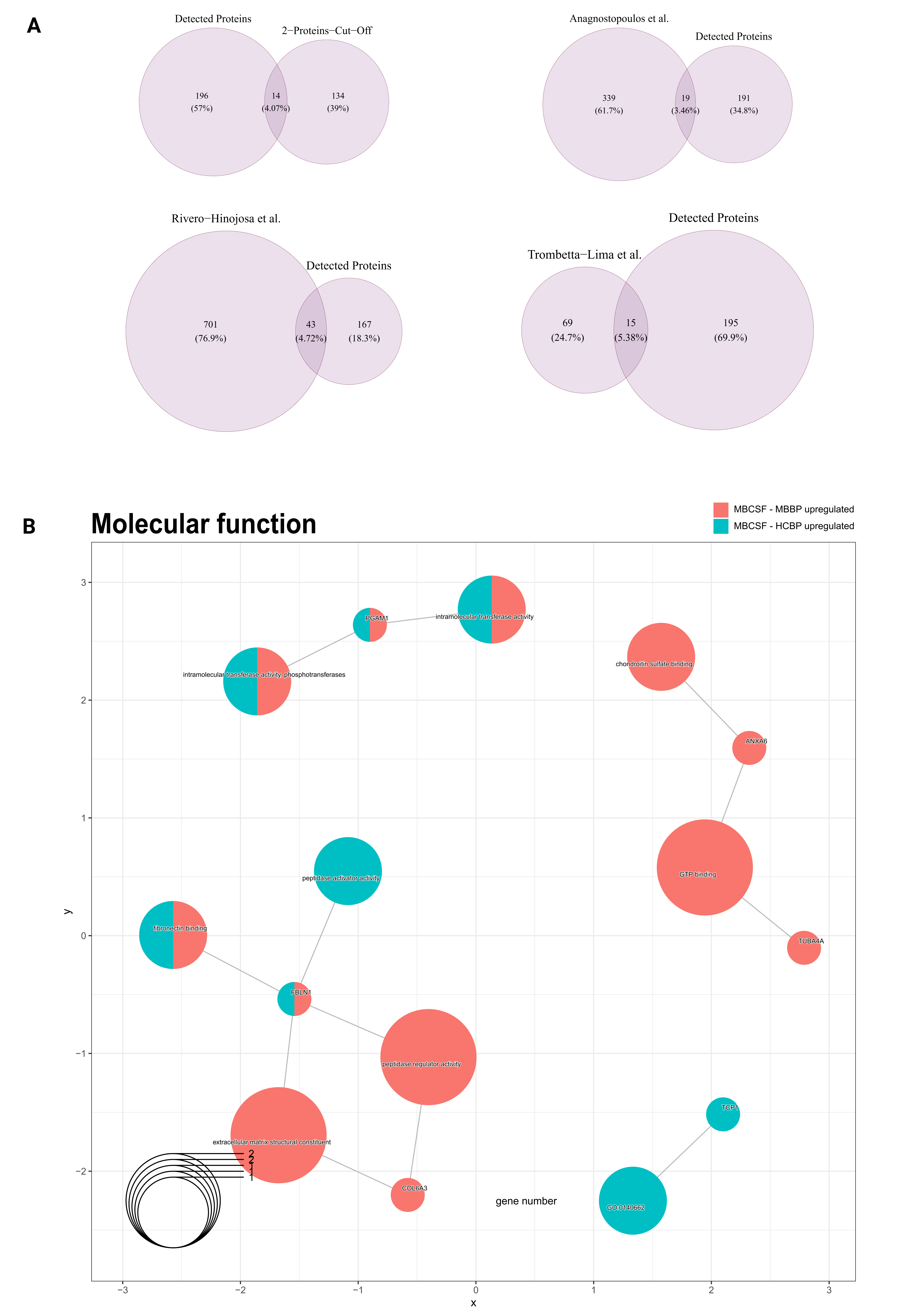

Supplement: Supplementary file 1 [file ijms-26-09279-s001.zip › 20 - Figure S20.png]

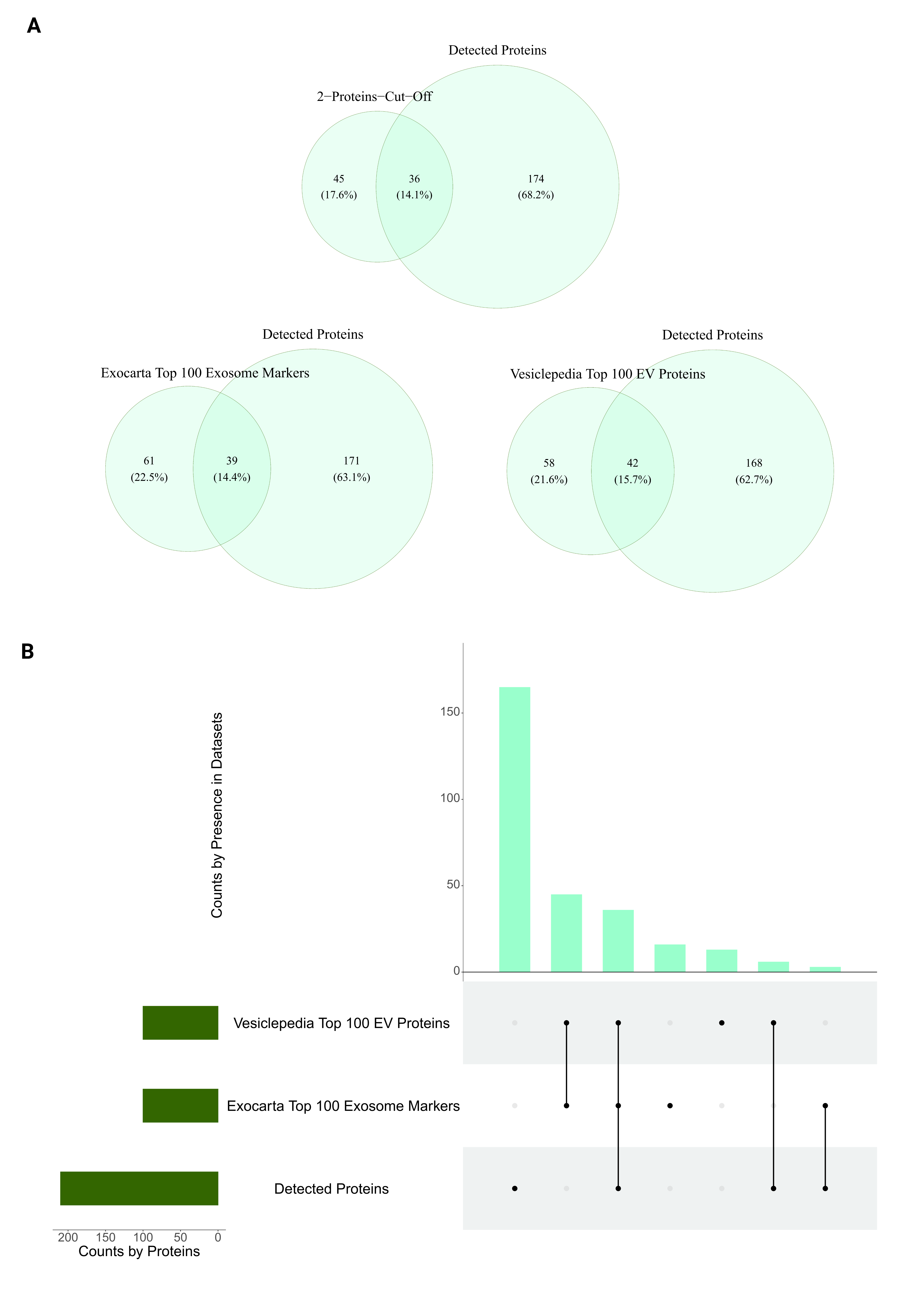

Supplement: Supplementary file 1 [file ijms-26-09279-s001.zip › 21 - Figure S21.png]

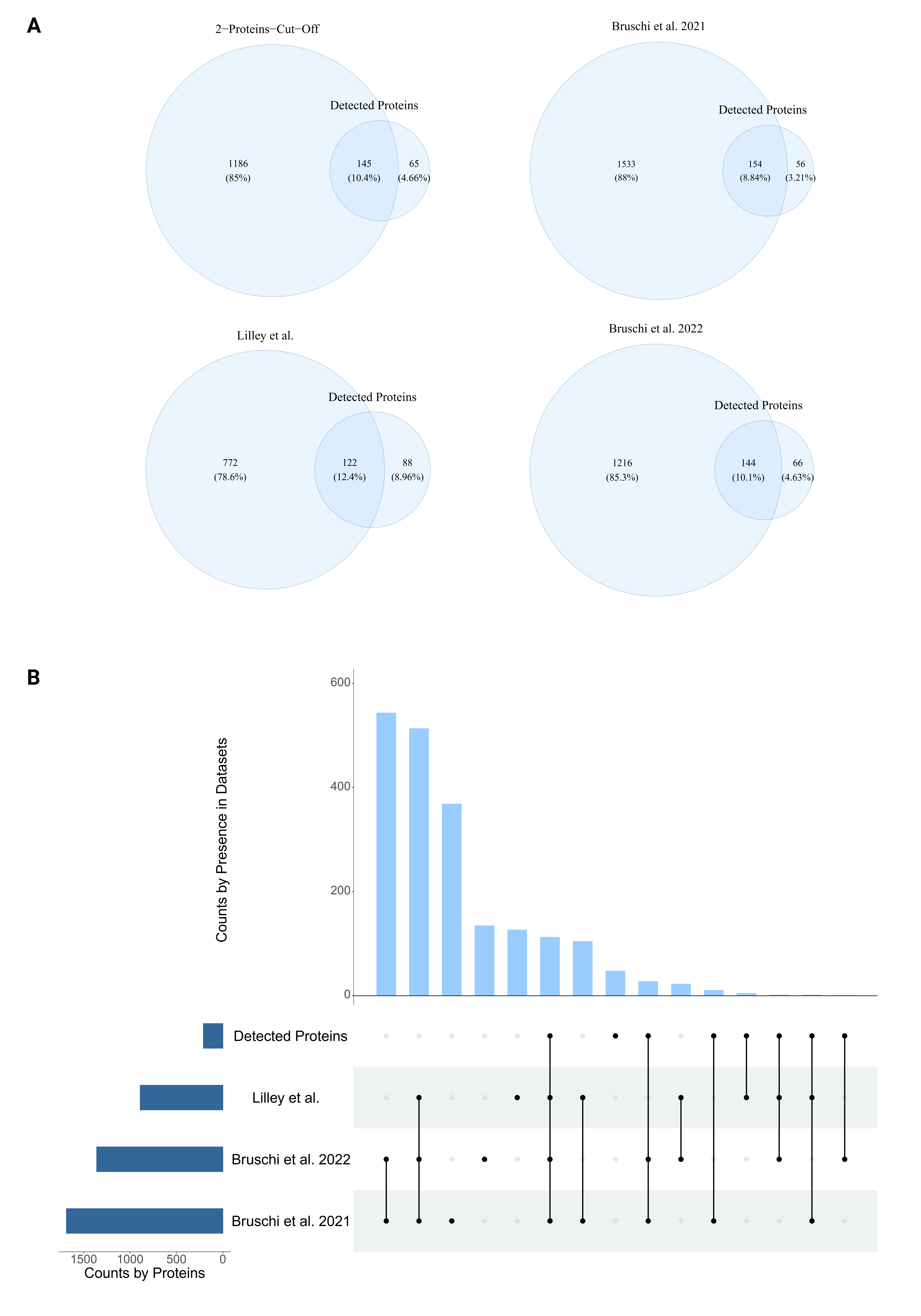

Supplement: Supplementary file 1 [file ijms-26-09279-s001.zip › 22 - Figure S22.png]

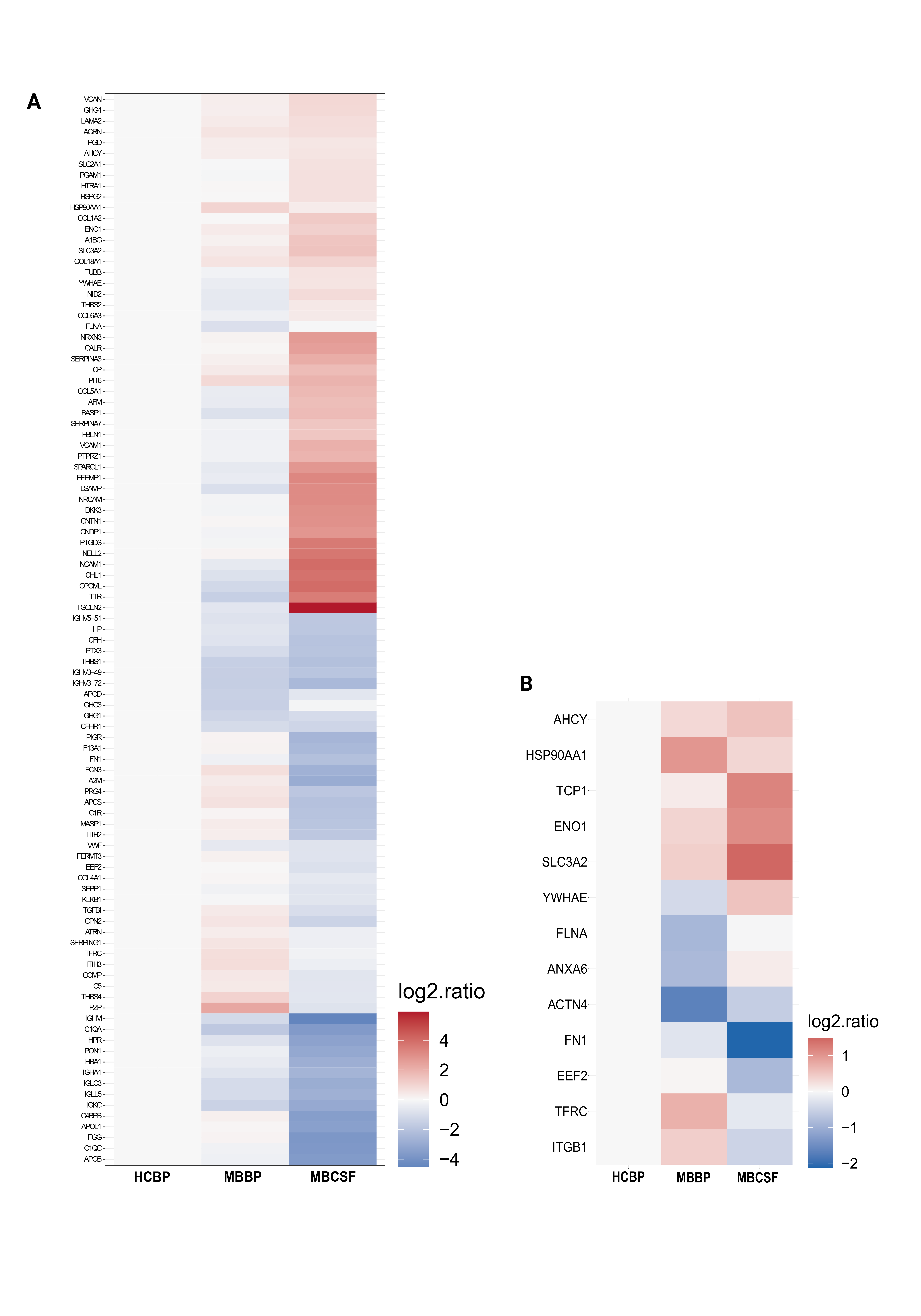

Supplement: Supplementary file 1 [file ijms-26-09279-s001.zip › 23 - Figure S23.png]

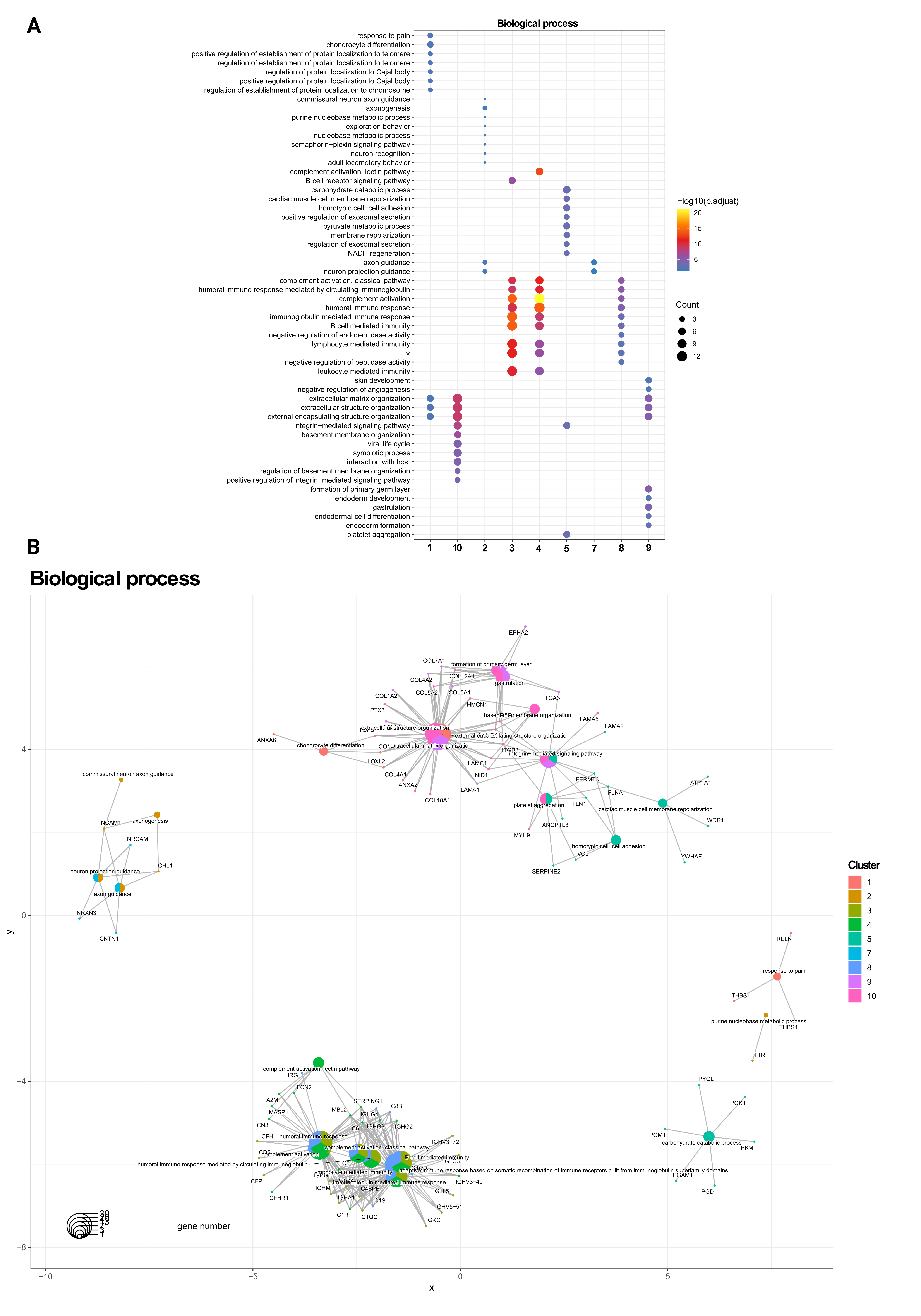

Supplement: Supplementary file 1 [file ijms-26-09279-s001.zip › 3 - Figure S3.png]

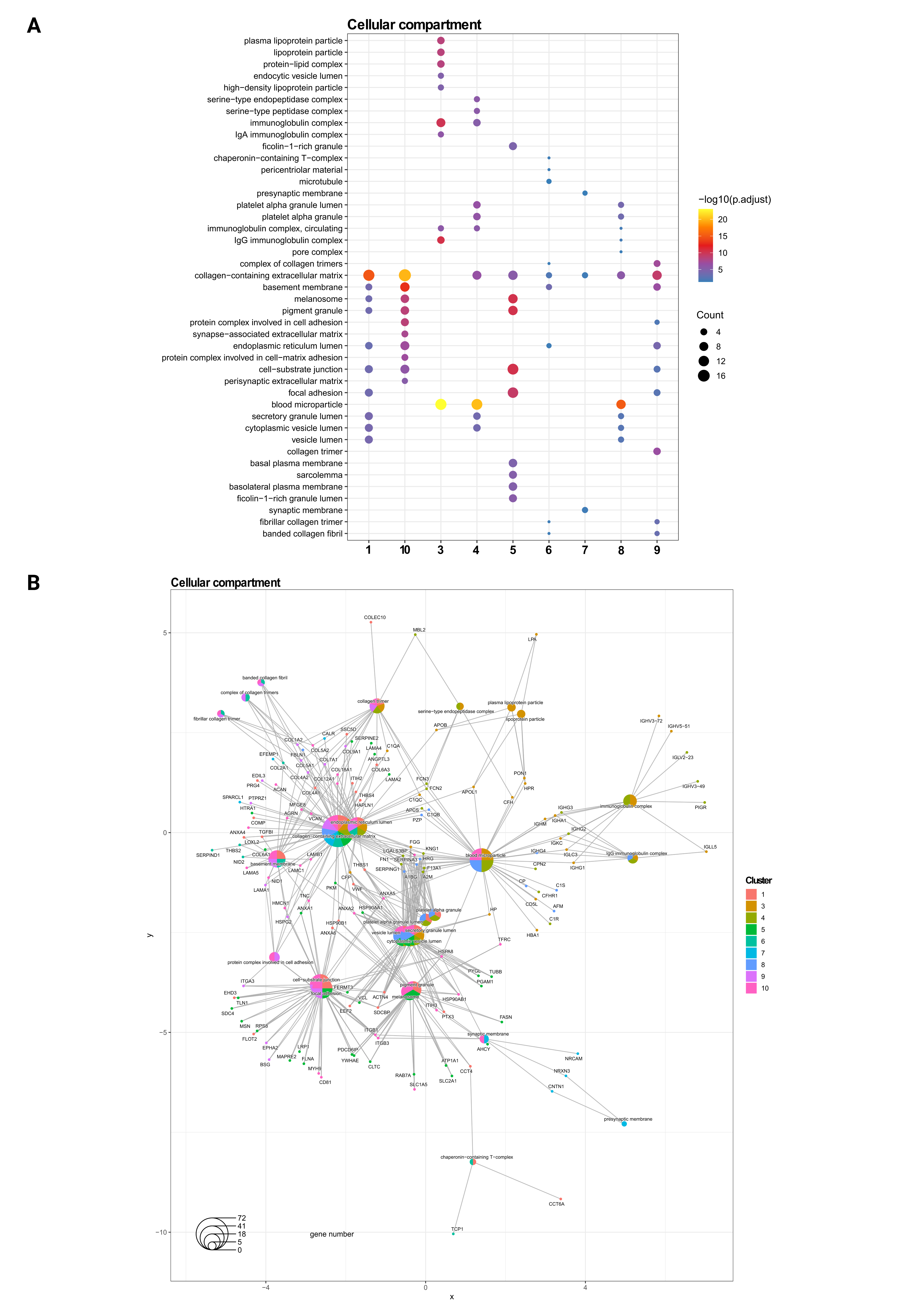

Supplement: Supplementary file 1 [file ijms-26-09279-s001.zip › 4 - Figure S4.png]

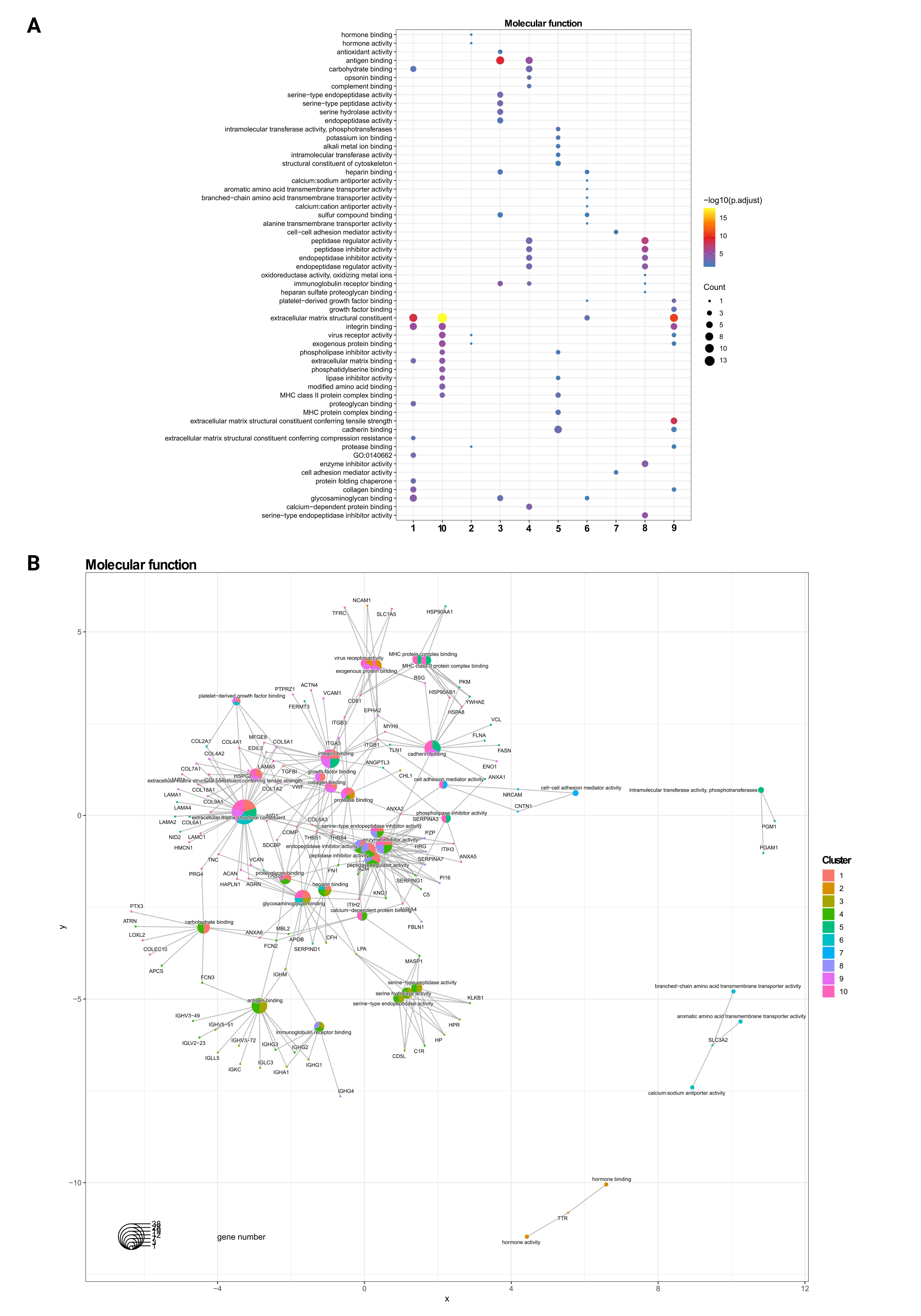

Supplement: Supplementary file 1 [file ijms-26-09279-s001.zip › 5 - Figure S5.png]

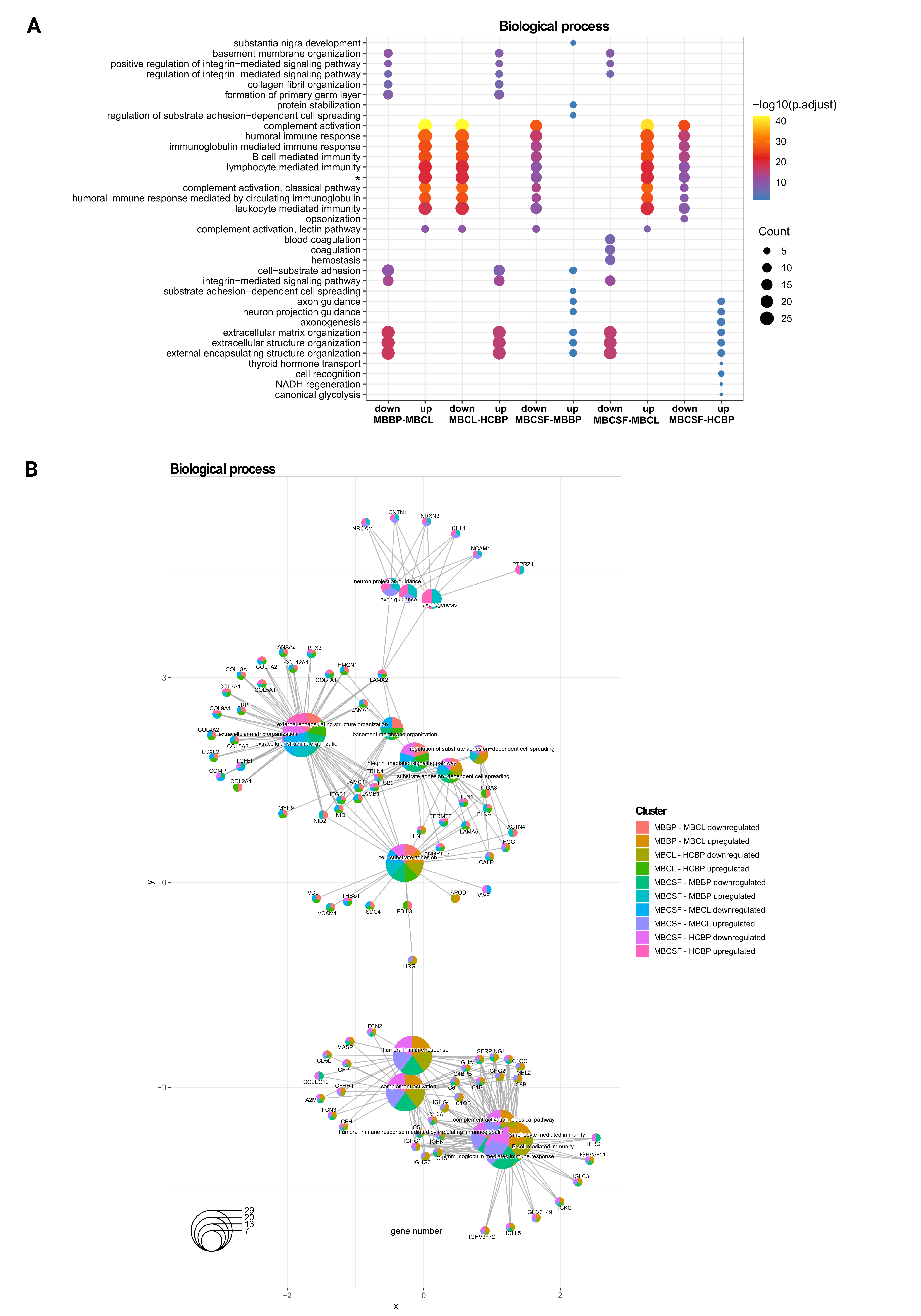

Supplement: Supplementary file 1 [file ijms-26-09279-s001.zip › 6 - Figure S6.png]

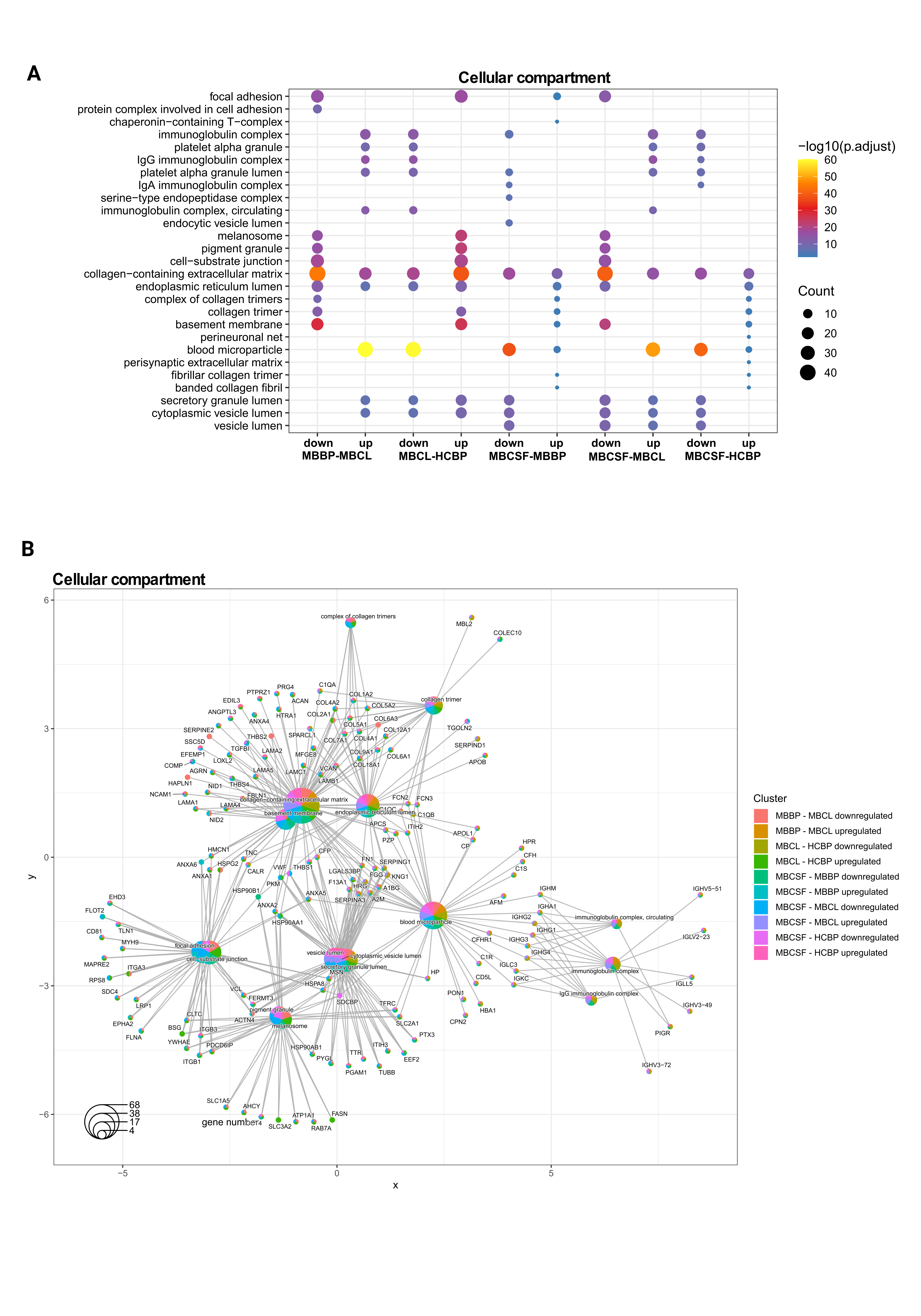

Supplement: Supplementary file 1 [file ijms-26-09279-s001.zip › 7 - Figure S7.png]

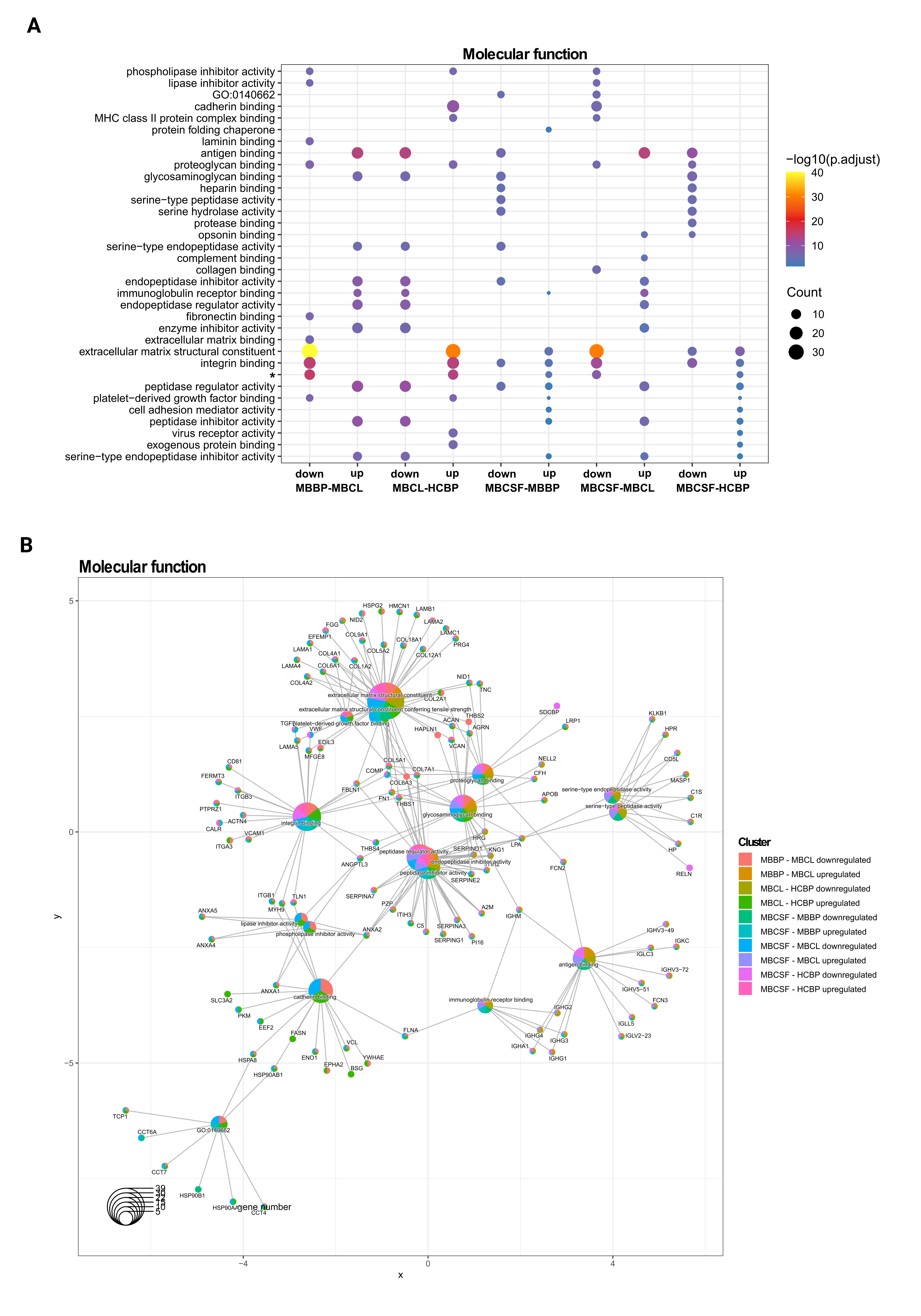

Supplement: Supplementary file 1 [file ijms-26-09279-s001.zip › 8 - Figure S8.png]

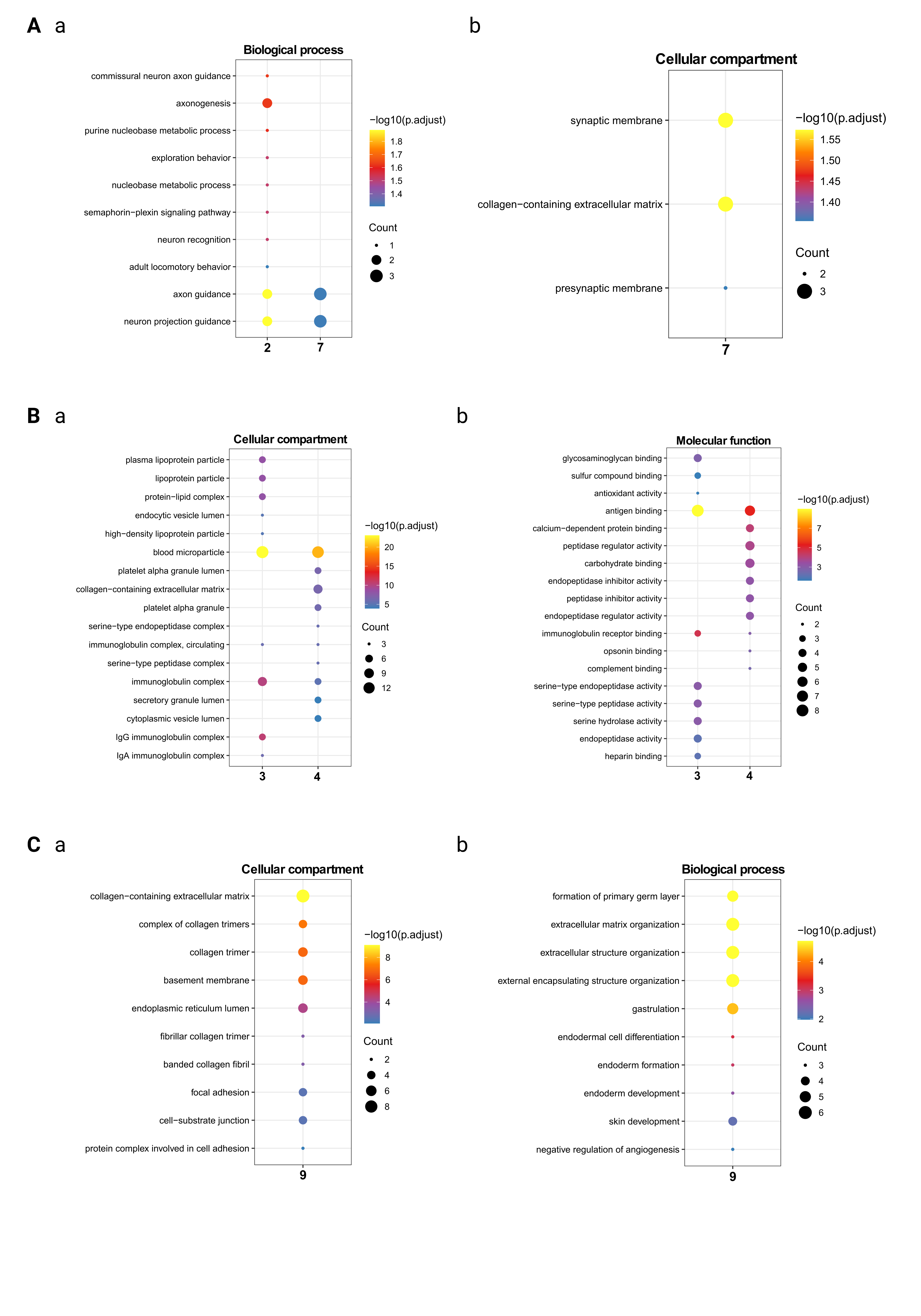

Supplement: Supplementary file 1 [file ijms-26-09279-s001.zip › 9 - Figure S9.png]
